# The Nobilamides: Potent Biofilm Inhibitors Produced by the Microbiota of Moon Snail Egg Masses

**DOI:** 10.1021/acsomega.5c01534

**Published:** 2025-06-20

**Authors:** Lois Kyei, Rose Campbell, Carla Menegatti, Emily Mevers

**Affiliations:** Department of Chemistry, 1757 Virginia Tech, 1040 Drillfield Dr. MC0212, Blacksburg, Virginia 24061, United States

## Abstract

Bacterial biofilm infections have become increasingly challenging to treat as bacteria living in a biofilm state are more resistant to antibiotics and protected from the host immune response. Eradicating biofilm infections generally requires treatment with high doses of antibiotics for prolonged periods; however, the rise in antibiotic resistance further challenges these treatments. Unfortunately, there are no approved drugs that inhibit or disrupt biofilm formation. Here, we leveraged our library of bacteria associated with moon snail egg masses found in Puerto Rico, using mass spectrometry-based metabolomics, to discover biofilm inhibitors. Analysis of a chemical fraction library revealed a set of peptides in fractions exhibiting potent inhibition of *Staphylococcus aureus* biofilms. Bioassay-guided isolation led to the isolation of lipopeptides, the nobilamides, which were previously shown to possess antibacterial activity and TRPV1 antagonist properties but were never evaluated in a biofilm inhibition assay. A thorough evaluation of the biofilm inhibition activity of A-3302-B and A-3302-A revealed they potently inhibit biofilm formation with IC_50_ of 161 ± 85 and 598 ± 66 nM, respectively. Interestingly, nobilamide A and B, linear analogs, are 500-fold less active than their cyclic analogs.

## Graphical abstract

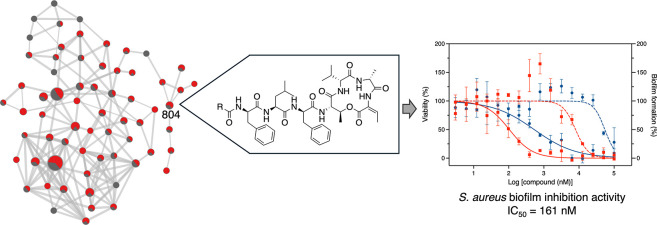

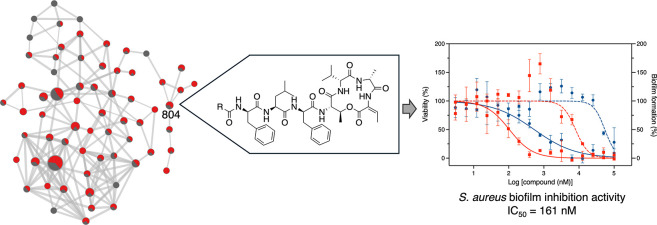

## Introduction

Bacterial infections are a growing cause of concern as current therapies lose efficacy and new pathogens continually emerge.^1–3^ The propensity of pathogens to form biofilms further challenges treatment options as bacteria living in a biofilm are up to 1000 times more resistant to current antibiotics.^4^ This is because bacteria growing in a biofilm are embedded in an extracellular polymeric substance (EPS) matrix containing polysaccharides, proteins, lipids, and DNA, protecting the bacteria from antibiotic exposure while impeding the host’s immune system.^5,6^ Biofilms develop when free-living planktonic cells transition to a sessile, aggregated lifestyle, a process controlled by various factors, including surface adhesion or nonsurface attached aggregation, environmental cues, and regulatory mechanisms such as quorum sensing.^7^ The aggregated cells generate the EPS to support the microcolony formation and biofilm maturation. Mature biofilm infections are particularly hazardous to those with implanted medical devices or with chronic disorders, such as Cystic Fibrosis.^8–10^ Although there is a critical need for the development of therapeutics to prevent or, even more importantly, disrupt mature biofilm growths, there are currently no approved therapies.

Many groups have attempted to develop small molecules that inhibit and/or disrupt preformed biofilms, and these efforts have been the focus of many recent review articles.^11–15^ We have also previously shown that bacteria associated with moon snail (*Neverita delessertiana*) egg collars from Florida are a good source of small molecule biofilm inhibitors against two ESKAPE pathogens, *Pseudomonas aeruginosa* and *Staphylococcus aureus*.^16^ The central hypothesis was that marine egg masses partner with bacteria to protect the host from biofouling. This led to the discovery that pseudochelin A, a previously known siderophore, inhibits *S. aureus* biofilm formation and was found within 62% of all active fractions. However, like many other biofilm inhibitors, pseudochelin A lacked potency (IC_50_ 88.5 μM) and was determined to act through sequestration of soluble iron, a mechanism unlikely to translate to efficacy in a mammalian host.

Continuing our efforts to discover natural products with potent biofilm inhibition properties, we used an integrative approach to investigate the bacteria associated with moon snail egg masses collected in Puerto Rico. Overlaying biological activity onto the Global Natural Product Social (GNPS) molecular networking output led to the discovery of a large family of cyclic and linear lipopeptides (Figure 1), including A-3302-B [also known as TL-119 (**1**)], A-3302-A (**2**), and nobilamides A (**3**) and B (**4**).^17–20^ The nobilamides have previously been reported to have a range of biological activities, including antibacterial properties,^18^ inhibiting cancer cell motility,^19^ long-acting antagonists of TRPV channels,^17^ inhibiting HSV-2 infections, and promoting biofilm formation in phytopathogenic and nonphytopathogenic strains,^20,21^ but had previously never been shown to have antibiofilm properties. Here we report the discovery that the cyclic lipopeptides exhibit potent (IC_50_ 161 ± 85 nM; **1**) inhibition of the formation of *S. aureus* biofilms, whereas their linear analogs exhibited no or minimal biofilm inhibition at concentrations up to 25 μM.

**Figure 1:**
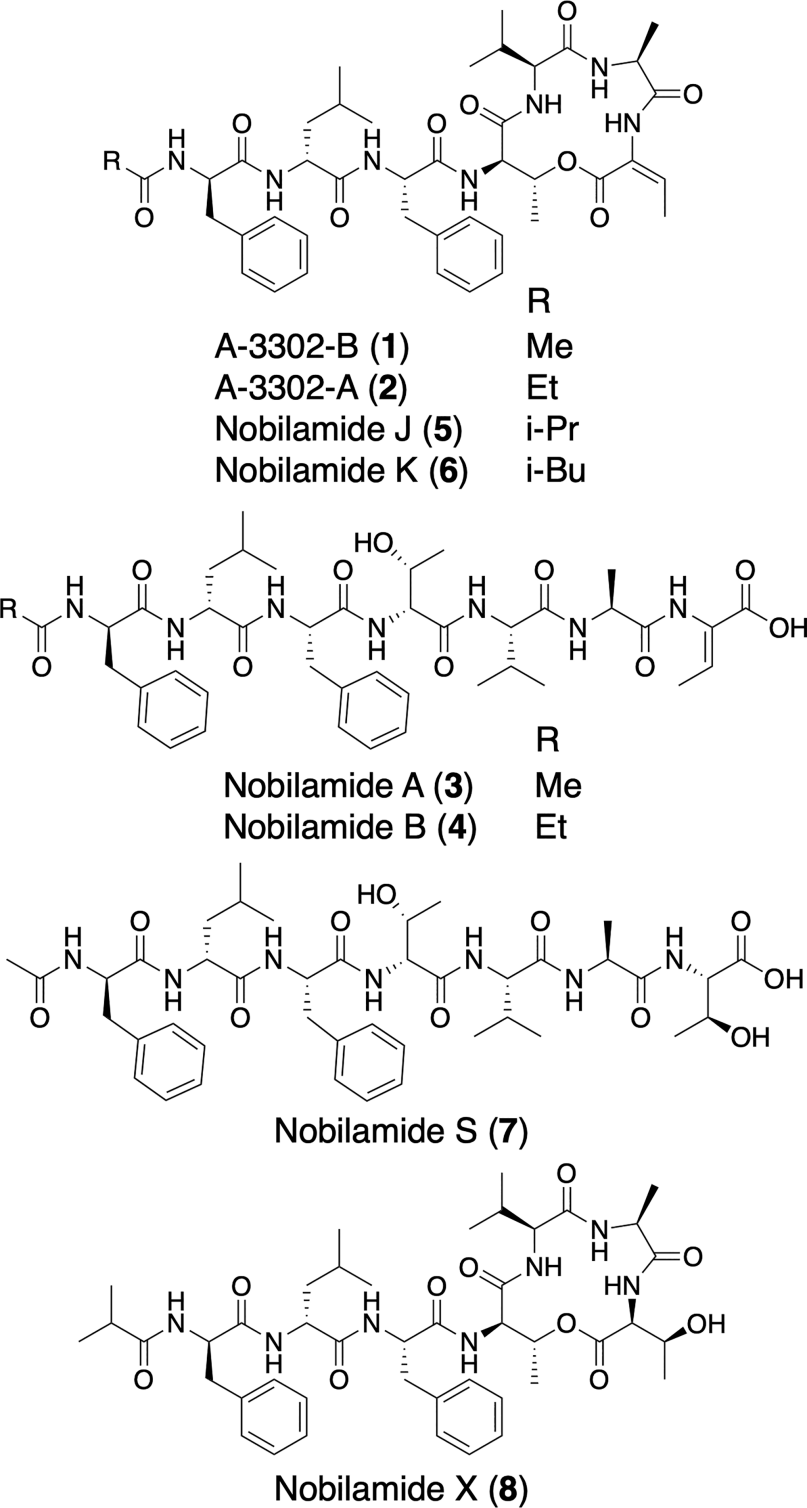
Structures of new and known *N*-acyl-heptapeptides.

## Results and Discussion

Moon snail egg masses were collected at Combate Beach in SW Puerto Rico, and fifty-three associated bacterial strains were isolated using various media conditions (Table S1). Each isolated bacterial strain was grown under three distinct media conditions (A, R2A, and YEME) with hydrophobic resins to capture secreted natural products (Table S2). The resins from each media condition were filtered and extracted using organic solvents to generate a single crude extract per bacterial strain. Each crude extract was semipurified based on polarity using C18 solid phase extraction (SPE) cartridges to yield two refined fractions. These refined fractions were analyzed by high-resolution liquid chromatography–mass spectrometry (HR-LCMS). At the same time, the crude extracts were screened for biofilm inhibition against both *S. aureus* and *P. aeruginosa*, with six and two fractions inhibiting >50% of biofilms, respectively (Figure S1).

The tandem MS spectra were analyzed using GNPS molecular networking (Figure S2), revealing a large spectral family of ions highly represented in active fractions.^22^ This subnetwork contained 65 nodes with precursor masses ranging from 632 to 920 *m*/*z* (Figure 2A). Dereplication of individual nodes using AntiBase, NPAtlas,^23^ and SciFinder initially led to the identification of four known compounds (**1**–**4**; Figure 1).^17,18^ Compounds **1**–**4** have previously been reported to have various biological activities including antibacterial properties,^18^ inhibiting cancer cell motility,^19^ long-acting antagonists of TRPV channels,^17^ inhibiting HSV-2 infections, and promoting biofilm formation against phytopathogenic and nonphytopathogenic bacterial strains^20,21^ but had previously never been shown to have antibiofilm properties. Therefore, the most abundant compounds in this subnetwork were prioritized for purification by high-performance liquid chromatography (HPLC). Nodes within the subnetwork are produced by five distinct bacterial strains: an *Exiguobacterium* sp. (EM729), a *Bacillus* sp. (EM740), two *Staphylococcu*s sp. (EM737 and EM762), and an unknown species (EM739). This represents five parent fractions with *S. aureus* biofilm inhibition activity (Table S1). EM758 was the only fraction with biofilm formation activity not present in this subnetwork, and manual analysis of the LCMS data confirmed that none of the identified masses were detectable.

**Figure 2:**
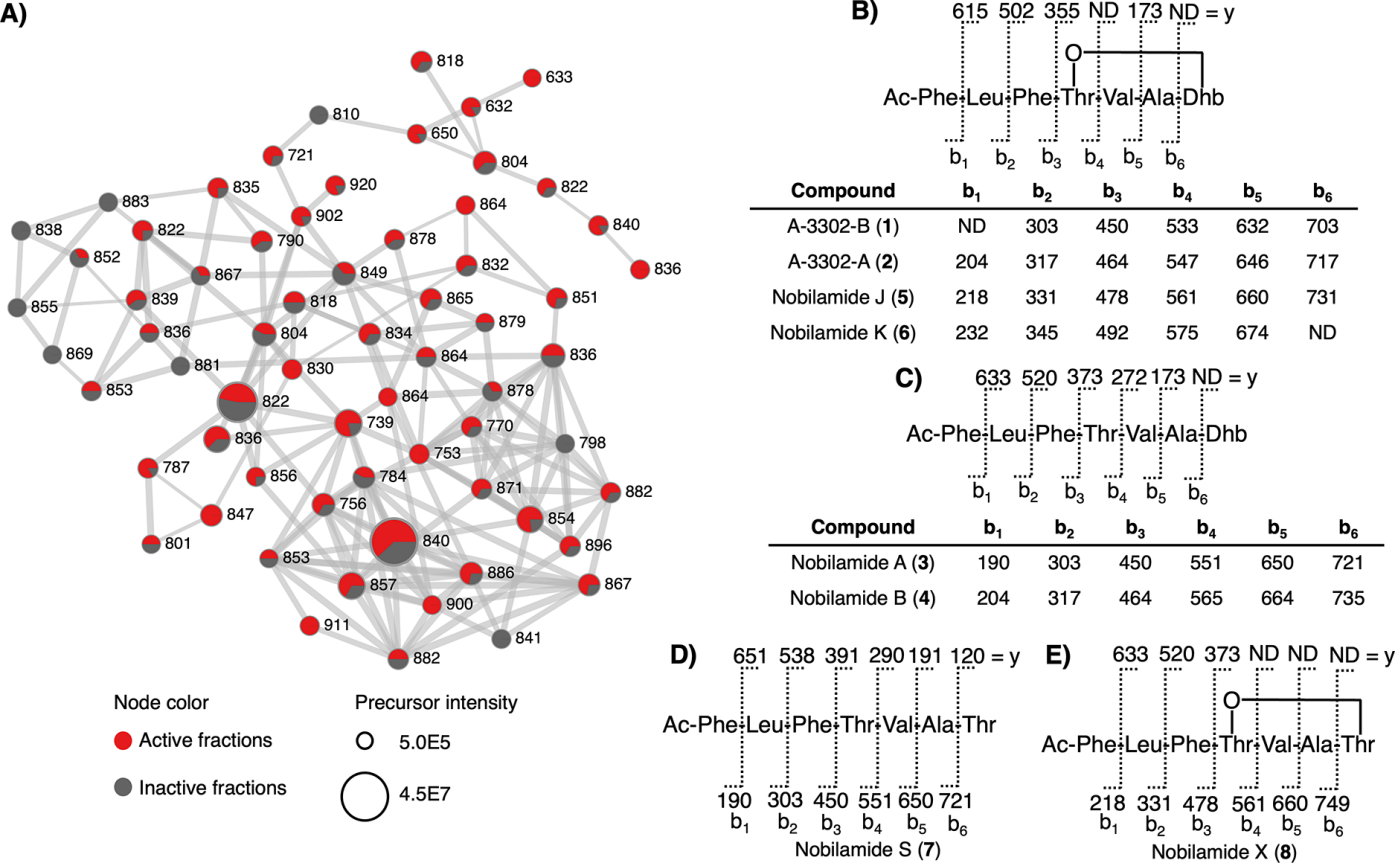
(A) Spectral family containing the nobilamides with nodes labeled with precursor masses and edge width representing cosine score (0.7–1.0), (B) tandem MS fragments of the cyclic peptides **1**, **2**, **5**, and **6**, (C) tandem MS fragments of the linear peptides **3** and **4**, (D) tandem MS fragments of nobilamide S with a threonine residue replacing Dhb, and (E) tandem MS fragments of nobilamide X (**8**). ND = not detected.

The fractions representing EM729 were chosen for further purification by reverse-phase HPLC as they appeared to contain larger quantities of many of the targeted compounds. Further purification of the nonpolar SPE fraction (100% MeOH) yielded the isolation of eight peptides (**1**–**8**). The NMR and MS data for **1**–**4** matched previously published data for A-3302-B [also known as TL-119 (**1**)], A-3302-A (**2**), nobilamides A (**3**) and B (**4**), respectively (Figures 1 and S3–S9).^17,18^ In addition, Marfey’s analysis on **1** matched the composition from the previous reports, indicating incorporation of l-Ala, l-Val, d-*allo*-Thr, l- and d-Phe, and d-Leu (Figures S10–S14). The two Phe were previously assigned through total synthesis of **1**,^20^ and the strong similarities in ^13^C NMR chemical shifts (Table S3) support the conclusion that our isolated material retains the same absolute configuration. All the lipopeptides are presumed to derive from the same biosynthetic pathway, and therefore, would be expected to incorporate amino acids with the same absolute configurations.

Tandem MS spectra for **5**–**8** confirmed these compounds were related to the nobilamides but, unfortunately, low isolated quantities prevented characterization by NMR. However, fragmentation data showed that **5**–**8** were highly similar to **1**–**4**; therefore, structural elucidation was accomplished by detailed analysis of the tandem MS spectra (Figures S15–S18). First, compounds **5** and **6** fragmented most similar to **1**, but their nominal masses differed by 28 and 42 Da, respectively. HRMS indicated that the structural differences were the addition of C_2_H_4_ to **5** and C_3_H_6_ to **6**. Detailed analysis of the tandem MS spectra revealed that all y-ions matched those observed in **1**, but the fragments representing b-ions were shifted by the observed difference in the precursor mass (Figure 2B,C). This indicated that **5** and **6** were cyclic *N*-acyl-heptadepsipeptides but incorporated butyl and pentyl lipids, respectively. The fragmentation for **7** was most similar to **3**, a linear version of **1**, but the nominal mass was 18 Da larger, indicating the inclusion of H_2_O. All b-ions matched those in **3**, but the fragments corresponding to the y-ions were 18 Da larger (Figure 2D). Based on a review by Wang et al. on the formation of dehydrobutyrine (Dhb) residues from threonine, the final amino acid loaded may be threonine, which subsequently undergoes dehydration to form Dhb.^24^ Compound **7** was proposed as a shunt product in which threonine was offloaded before undergoing dehydration. Analysis of the fragmentation pattern of **7** confirmed it contained an unmodified threonine in the terminal position, which was represented by a 120 *m*/*z* fragment ion. Dereplication of the elucidated structures revealed a recent study by Iloabuchi and Spiteller (2024) that reported the structures of 14 new nobilamide analogs, including **5**–**7**;^20^ which had yet to be added to any of the databases used for dereplication. Thorough NMR analysis by Iloabuchi and Spiteller confirmed the *N*-acyl tails in **5** and **6** were isopropyl and isobutyl lipids, respectively.

Finally, the fragmentation pattern for **8** was most similar to **5**, but its nominal mass is 18 Da larger. Analysis of the tandem MS spectra revealed that many b-ions matched those observed in **5**, but the fragments representing y-ions were shifted by the observed difference in the precursor mass (Figure 2E). This indicated that **8** was a cyclic lipopeptide and incorporated a threonine rather than Dhb, representing a new *N*-acyl-heptapeptide named nobilamide X.^17,19,20^ It is possible that the linear analogs are either formed directly through the use of water to off-load the mature peptide from the thioesterase or they could be artifacts of the isolation process.

Only **1**–**4** were isolated in sufficient quantities for biological evaluation of biofilm inhibition properties. Both cyclic lipopeptides, **1** and **2**, exhibited antibacterial activity against *S. aureus*, similar to previous reports, with MICs of 25 and 100 μM, respectively.^25^ Interestingly, at subinhibitory levels, both potently (IC_50_ 161 ± 85 and 598 ± 66 nM, respectively) inhibited biofilm formation without impeding bacterial growth (Figures 3, S19 and S20). This concentration-dependent change in activity profile has been previously reported for other antibiotics, including tobramycin, tetracycline, and norfloxacin with each functioning as signaling molecules at low concentrations and as antibacterial agents at high concentrations.^26^ Although several nobilamides have previously been reported to promote biofilm formation in select phytopathogenic and nonphytopathogenic bacterial strains, our recent findings demonstrate that they strongly inhibit *S. aureus* biofilms. This divergence could be a result of the differences in species-specific responses induced by the compounds across bacterial species. For instance, subinhibitory concentrations of furanones have been reported to inhibit *P. aeruginosa* biofilm formation while promoting biofilm formation in *S. aureus* *.* ^27,28^

**Figure 3:**
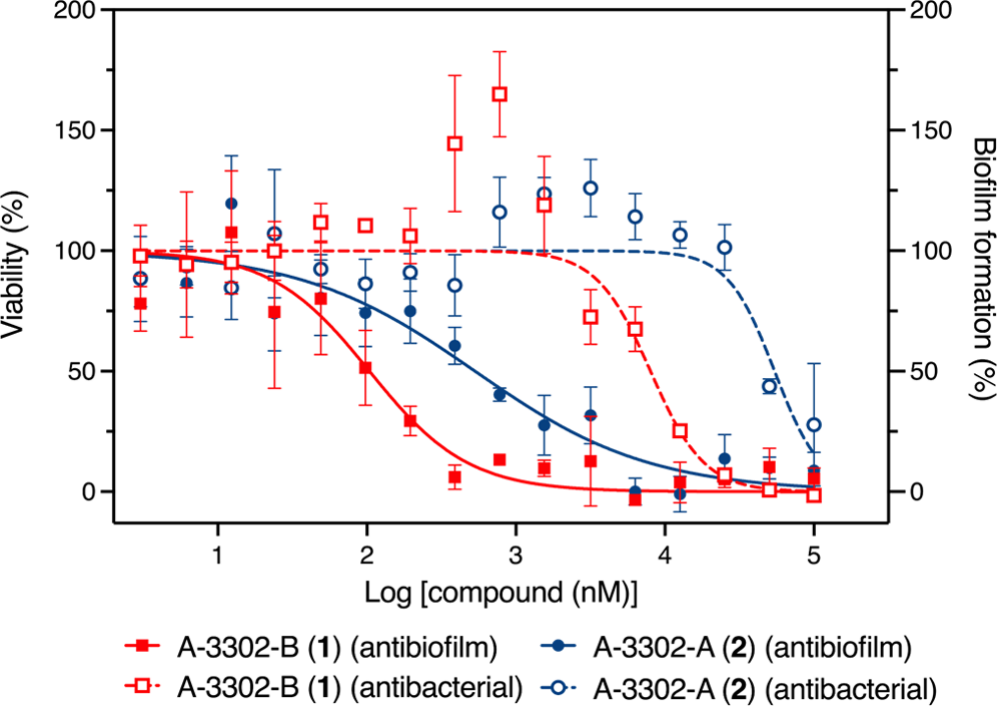
Antibiofilm (solid line) and antibacterial (dashed line) activities of the cyclic depsipeptides A-3302-B (**1**) (red squares) and A-3302-A (**2**) (blue circles) against*S. aureus*.

The potency, particularly of **1**, is significantly improved compared to our previous study^16^ and is within range of some of the most potent biofilm inhibitors, including carolacton, a bacterial polyketide that inhibits 35% of *Streptococcus mutans* biofilms at 10 nM.^29^ Conversely, the linear peptides **3** and **4** were significantly less active, with no antibacterial activity or inhibition of biofilm formation at concentrations up to 25 μM (Figures S21 and S22). The stark activity differences between the linear and cyclic peptides indicate the importance of the macrocycle for activity. In addition, **1** is roughly five times more potent than **2**, suggesting the extension in the lipid chain is also not well tolerated. Compounds **1** and **2** were further evaluated in biofilm disruption assays to evaluate their ability to disrupt preformed biofilms; however, they showed no significant biofilm disruption activity at concentrations below 50 μM. This suggests that the observed antibiofilm activity is specific to the early stages of biofilm development rather than acting as a biofilm disruption agent (Figures S23 and S24). Unfortunately, we did not have enough material to attempt to identify the mechanism of action of **1** and **2** in inhibiting the early stages of biofilm development. However, several peptides with biofilm inhibition properties have been reported to disrupt the membrane of biofilm-embedded cells (nisin A),^30^ downregulate genes promoting quorum sensing (human host defense peptide LL-37)^31^ or degrade the EPS matrix (hepcidin 20).^32^

The discovery of potent inhibitors of biofilm formation by the bacterial-derived nobilamides adds to the growing knowledge of an unusual class of lipopeptides, where macrocyclization involves the side chain of one of the amino acid residues. Structurally, these compounds likely derive from a hybrid polyketide synthase (PKS)/nonribosomal peptide synthetase (NRPS), yet the gene cluster has yet to be identified. Although many analogs are described, the main structural differences involve the incorporation of different lipid moieties with some slight changes in the amino acid composition. However, the nobilamides are related to many other cyclic lipopeptides that have incorporated cyclization between the C-terminus and the side chain of an amino acid rather than the *N*-terminus, including the tiglicamides,^33^ xentrivalpeptides,^34^ cyanogripeptides,^35^ lyngbyastatins,^36^ molassamides,^37^ seongsanamides,^38^ and bouillomides.^39^ Interestingly, these natural products are commonly produced by both cyanobacteria and pathogenic bacteria, with many of these molecules being reported to have a diverse range of biological activity. Therefore, this family of compounds is likely to play a role in shaping the local environment.

## Conclusion

In summary, chemical investigations into the natural product potential of bacteria associated with moon snail egg masses collected in Puerto Rico revealed a family of lipopeptides that possess potent biofilm inhibition activity against *S. aureus*. Overlaying the metabolomic analysis with biological activity indicated that a single subnetwork was responsible for the observed biofilm inhibition. The structures of each lipopeptide were characterized using tandem mass spectrometry. Interestingly, the cyclic lipopeptides, A-3302-B and A-3302-A, demonstrated potent inhibition of *S. aureus* biofilm formation, with IC_50_ values of 161 ± 85 and 598 ± 66 nM, respectively. In contrast, the linear lipopeptides nobilamides A and B were inactive at concentrations up to 25 μM, emphasizing the critical role of the macrocycle for bioactivity. Our findings underscore the significance of marine egg mass-associated bacterial metabolites as a promising source of biofilm inhibitors.

## Methods

### General Experimental Procedures

NMR spectra were recorded in deuterated methanol with the residual solvent peak as an internal standard (δ_C_ 49.00, δ_H_ 3.31) on a Bruker AVANCE III 600 MHz instrument equipped with a triple resonance inverse (CP-TCI) Prodigy N2 cooled CryoProbe (600 and 150 MHz for ^1^H and ^13^C NMR, respectively). LR-LCMS data was obtained on an Agilent 1200 series HPLC system equipped with a photodiode array detector and a Thermo LTQ mass spectrometer. HR-ESI-MS was carried out on either a Shimadzu q-ToF mass spectrometer equipped with an HPLC system or a Thermo Q Exactive Plus mass spectrometer equipped with an electrospray ionization (ESI) source coupled with a Waters Acquity ultraperformance liquid chromatography. HPLC purifications were carried out on Agilent 1200 series or 1260 Infinity II HPLC systems (Agilent Technologies) equipped with a photodiode array detector. All solvents were of HPLC quality. Microplate readings were taken on the Byonoy absorbance 96 microplate reader.

### Bacterial Isolation from Moon Snail Egg Masses Collected in Puerto Rico

Egg collars for isolation of bacterial strains were collected from one site in SW Puerto Rico in December 2021 (GPS coordinates: 17.978961, −67.213472). Bacterial strains were isolated and preserved using methods previously described.^16^

### Bioassay-Guided Isolation of Natural Products

*Exiguobacterium* sp. (EM729) was grown in 50 mL cultures using A, R2A, and YEME with 30 g/L of hydrophobic resins (15 g/L of HP-20, 7.5 g/L of XAD4 and XAD7) for 10 d at 30 °C, 150 rpm (Table S2). Resins were filtered using miracloth and extracted with acetone (4 h, rt) and CH_3_OH (16 h, rt). The organic material was filtered through a coffee filter and dried under vacuum. The dried crude extract was fractionated in a step-gradient using a 5 g C8 reversed-phase SPE to generate two fractions: 50% CH_3_OH/H_2_O (fraction A) and 100% CH_3_OH (fraction B) using 30 mL of each eluent. Fraction B (10 mg) contained the target compounds and was thus chosen for further purification by HPLC. HPLC equipped with a Phenomenex Synergi 4 μm Hydro-RP C18 (250 × 10 mm) column with a flow rate of 3 mL/min, held at 40% CH_3_CN + 0.1% formic acid (FA)/60% H_2_O + 0.1% FA for 5 min then gradient to 60% CH_3_CN + 0.1% FA/40% H_2_O + 0.1% FA over 55 min; (**1**) 2.6 mg, (**2**) 0.5 mg, (**3**) 0.4 mg, (**4**) 0.4 mg, (**5**) 0.2 mg, (**6**) 0.3, (**7**) 0.3 mg, (**8**) 0.1 mg. All attempts to cultivate EM729 and other producing strains in large cultures (>50 mL) led to the loss of production of the targeted metabolites.

#### A-3302-B (**1**)

Pale yellow amorphous solid; ^1^H NMR (CD_3_OD, 600 MHz): δ_H_ 7.28 (m, 2H), 7.27 (m, 2H), 7.21 (m, 2H), 7.17 (m, 2H), 7.09 (m, 2H), 6.82 (q, 1H, *J* = 7.1 Hz), 4.67 (t, 1H, *J* = 5.1 Hz), 4.63 (br s, 1H), 4.47 (q, 1H, *J* = 7.5 Hz), 4.18 (d, 1H, *J* = 10.7 Hz), 4.06 (br s, 1H), 4.04 (br s, 1H), 3.97 (dd, 1H, *J* = 7.2, 8.8 Hz), 3.43 (m, 1H), 3.10 (dd, 1H, *J* = 5.8, 13.8 Hz), 3.00 (m, 1H), 2.84 (dd, 1H, *J* = 4.9, 13.8 Hz), 2.24 (m, 1H), 2.11 (s, 3H), 1.68 (d, 3H, *J* = 7.2 Hz), 1.45 (d, 3H, *J* = 7.4 Hz), 1.43 (m, 1H), 1.29 (m, 3H), 1.27 (m, 1H), 1.01 (d, 3H, *J* = 6.4 Hz), 0.99 (d, 3H, *J* = 6.4 Hz), 0.98 (m, 1H), 0.83 (d, 3H, *J* = 6.5 Hz), 0.73 (d, 3H, *J* = 6.6 Hz). ^13^C­{1H} NMR (CD_3_OD, 125 MHz): δ_C_ 175.1, 173.3 × 2, 173.2, 165.4, 139.0, 137.7, 137.2, 131.3 × 2, 130.3 × 2, 129.4 × 2, 128.8 × 2, 127.6 × 2, 126.7, 74.1, 62.8, 59.8, 56.1, 54.5, 51.5, 40.5, 38.7, 37.9, 30.5, 24.9, 23.0, 22.7, 22.4, 19.8, 19.4, 17.3, 17.2, 15.1 (four carbonyl carbons are missing due to peak broadening); ESI MS/MS (Orbitrap) *m*/*z*: 703.3858 (C_38_H_51_N_6_O_7_), 632.3485 (C_35_H_46_N_5_O_6_), 615.3508 (C_31_H_47_N_6_O_7_), 533.2725 (C_30_H_37_N_4_O_5_), 502.2629 (C_25_H_36_N_5_O_6_), 450.2389 (C_26_H_32_N_3_O_4_), 355.1988 (C_16_H_27_N_4_O_5_), 303.1705 (C_17_H_23_N_2_O_3_), 173.0932 (C_7_H_13_N_2_O_3_); HRMS (ESI) *m*/*z*: [M + H]^+^ calcd for C_42_H_59_N_7_O_9_^+^, 804.4291; found, 804.4295.

#### A-3302-A (**2**)

Pale yellow amorphous solid; ESI MS/MS (Orbitrap) *m*/*z*: 717.3901 (C_39_H_53_N_6_O_7_), 646.3552 (C_36_H_48_N_5_O_6_), 615.3547 (C_31_H_47_N_6_O_7_), 547.2887 (C_31_H_39_N_4_O_5_), 502.2626 (C_25_H_36_N_5_O_6_), 464.2580 (C_27_H_34_N_3_O_4_), 355.1988 (C_16_H_27_N_4_O_5_), 317.1847 (C_17_H_23_N_2_O_3_), 204.1038 (C_12_H_14_NO_2_), 173.1276 (C_7_H_13_N_2_O_3_); HRMS (ESI) *m*/*z*: [M + H]^+^ calcd for C_43_H_60_N_7_O_9_^+^, 818.4447; found, 818.4446.

#### Nobilamide A (**3**)

ESI MS/MS (Orbitrap) *m*/*z*: 721.3971 (C_38_H_53_N_6_O_8_), 650.3523 (C_35_H_48_N_5_O_7_), 633.3570 (C_35_H_47_N_5_O_6_), 551.2908 (C_30_H_39_N_4_O_6_), 520.2834 (C_25_H_38_N_5_O_7_), 450.2398 (C_26_H_32_N_3_O_4_), 373.2124 (C_16_H_29_N_4_O_6_), 303.1703 (C_17_H_23_N_2_O_3_), 272.1616 (C_12_H_22_N_3_O_4_), 190.0864 (C_11_H_12_NO_2_), 173.1276 (C_7_H_13_N_2_O_3_); HRMS (ESI) *m*/*z*: [M + H]^+^ calcd for C_42_H_60_N_7_O_10_^+^, 822.4396; found, 822.4406.

#### Nobilamide B (**4**)

ESI MS/MS (Orbitrap) *m*/*z*: 735.4170 (C_39_H_55_N_6_O_8_), 664.3655 (C_36_H_50_N_5_O_7_), 633.3560 (C_31_H_49_N_6_O_8_), 565.3026 (C_31_H_41_N_4_O_6_), 520.2757 (C_25_H_38_N_5_O_7_), 464.2579 (C_27_H_34_N_3_O_4_), 373.2062 (C_16_H_29_N_4_O_6_), 317.1848 (C_18_H_25_N_2_O_3_), 272.1619 (C_12_H_22_N_3_O_4_), 204.1008 (C_12_H_14_NO_2_), 173.1278 (C_7_H_13_N_2_O_3_); HRMS (ESI) *m*/*z*: [M + H]^+^ calcd for C_43_H_62_N_7_O_10_^+^, 836.4553; found, 836.4556.

#### Nobilamide J (**5**)

ESI MS/MS (Orbitrap) *m*/*z*: 731.4040 (C_40_H_55_N_6_O_7_), 660.3742 (C_37_H_50_N_5_O_6_), 615.3556 (C_31_H_47_N_6_O_7_), 561.3091 (C_32_H_41_N_4_O_5_), 502.2626 (C_25_H_36_N_5_O_6_), 478.2648 (C_28_H_36_N_3_O_4_), 355.1984 (C_16_H_27_N_4_O_5_), 331.2021 (C_19_H_27_N_2_O_3_), 218.1176 (C_13_H_16_NO_2_), 173.1276 (C_7_H_13_N_2_O_3_); HRMS (ESI) *m*/*z*: [M + H]^+^ calcd for C_44_H_62_N_7_O_9_^+^, 832.4604; found, 832.4607.

#### Nobilamide K (**6**)

ESI MS/MS (Orbitrap) *m*/*z*: 674.4007 (C_38_H_52_N_5_O_6_), 615.3568 (C_31_H_47_N_6_O_7_), 575.3235 (C_33_H_43_N_4_O_5_), 502.2624 (C_25_H_36_N_5_O_6_), 492.2851 (C_29_H_38_N_3_O_4_), 355.1988 (C_16_H_27_N_4_O_5_), 345.2188 (C_20_H_29_N_2_O_3_), 232.1327 (C_14_H_18_NO_2_); HRMS (ESI) *m*/*z*: [M + H]^+^ calcd for C_45_H_64_N_7_O_9_^+^, 846.4760; found, 846.4760.

#### Nobilamide S (**7**)

ESI MS/MS (Orbitrap) *m*/*z*: 721.3981 (C_38_H_53_N_6_O_8_), 651.3725 (C_31_H_51_N_6_O_9_), 650.3678 (C_35_H_48_N_5_O_7_), 551.2917 (C_30_H_39_N_4_O_6_), 538.2824 (C_25_H_40_N_5_O_8_), 450.2401 (C_26_H_32_N_3_O_4_), 391.2252 (C_16_H_31_N_4_O_7_), 303.1706 (C_17_H_23_N_2_O_3_), 290.1725 (C_12_H_24_N_3_O_5_), 191.1022 (C_7_H_15_N_2_O_4_), 190.0849 (C_11_H_12_NO_2_), 120.0808 (C_4_H_10_NO_3_); HRMS (ESI) *m*/*z*: [M + H]^+^ calcd for C_42_H_62_N_7_O_11_^+^, 840.4502; found, 840.4503.

#### Nobilamide X (**8**)

ESI MS/MS (Orbitrap) *m*/*z*: 749.4223 (C_40_H_57_N_6_O_8_), 660.3742 (C_37_H_50_N_5_O_6_), 633.3569 (C_31_H_49_N_6_O_8_), 561.3079 (C_32_H_41_N_4_O_5_), 520.2707 (C_25_H_38_N_5_O_7_), 478.2745 (C_28_H_36_N_3_O_4_), 331.2016 (C_19_H_27_N_2_O_3_), 272.1628 (C_12_H_22_N_3_O_4_), 218.1173 (C_13_H_16_NO_2_); HRMS (ESI) *m*/*z*: [M + H]^+^ calcd for C_44_H_64_N_7_O_10_^+^, 850.4709; found, 850.4706.

### Bacterial Strains and Biofilm Inhibition Assay

The biofilm inhibition assay was performed as previously described by Kyei et al.^16^ Briefly, an aliquot (2 μL) of each refined fraction (5 M in DMSO) was added to 96-well plates in quadruplicates. DMSO was used as a negative control and alizarin (500 μM) was used as the positive control.^40^ An overnight culture of *S. aureus* (ATCC BAA-2313) grown in Luria–Bertani (LB) medium was diluted to a starting OD_600_ of 0.01 in M63 liquid broth, and 98 μL was transferred to each well. Inoculated plates were incubated in a static incubator for 24 h at 37 °C. The antibacterial effects of the fractions were determined by reading the OD of the plates at 595 nm. Planktonic cells were discarded, and the plates were washed 3 times with sterile Milli-Q water and dried for 30–60 min at rt. Adhered biofilms were stained with 115 μL of 0.1% (wt/vol) crystal violet (aq) and shaken at 60 rpm for 30 min. The plates were washed and dried (30–60 min) at rt, and then the crystal violet-bound biofilms were solubilized by the addition of 130 μL of 30% acetic acid (aq). The solubilized material was transferred to new 96-well plates, and absorbance was measured at 595 nm.

### Dose-Dependent Biofilm Inhibition Assay

Compounds **1**–**4** were dissolved in DMSO to stock concentrations of 5000 μM. The stock solutions for **1** and **2** were serially diluted (2-fold) to yield 16 concentrations (5000–0.15 μM), while those for **3** and **4** were only diluted to yield 8 concentrations (5000–39.06 μM) due to low isolated quantities. Compounds **1** and **2** were evaluated in the biofilm inhibition assay with four technical replicates and three biological replicates, while compounds **3** and **4** were tested with three technical replicates and two biological replicates. An aliquot (2 μL) of each concentration was used in the assay following the protocol described in the “Bacterial Strains and Biofilm Inhibition Assay” section above.

### Dose-Dependent Biofilm Disruption Assay

The biofilm disruption assay was performed as previously described by Kyei et al.^16^ Compounds **1**–**4** were dissolved in DMSO to stock concentrations of 5000 μM. The stock solutions for **1** and **2** were serially diluted (2-fold) to yield eight concentrations (5000–39.06 μM). *S. aureus* biofilms were first established overnight in 100 μL of M63 minimal media broth, and bacterial growth was measured at OD_600_ to ensure uniformity in growth across the plates. The spent media and planktonic bacteria were aspirated and discarded. The plates were gently rinsed with fresh media three times. Fresh media (108 μL) was added to the planets together with 2 μL of the stock concentrations prepared in quadruplicate. The plates were incubated under static conditions for 16 to 20 h at 37 °C. After incubation, OD_600_ was recorded to measure bacterial growth. The spent supernatant media was discarded, and the plates were rinsed (×3) with Milli-Q water and left for 30–60 min to air-dry at rt. The adhered biofilms were stained with 0.1% (wt/vol) crystal violet (aq) and placed on an orbital shaker at low speed for 30 min. Planktonic cells were washed, the plates were dried (30–60 min) at rt and then the crystal violet-bound biofilms were solubilized in 30% acetic acid (aq). The solubilized material was transferred to 96-well plates and then absorbance recorded at 595 nm.

### Acid Hydrolysis and Marfey’s Analysis of A-3302-B (**1**)

An aliquot (100 μg) of A-3302-B (**1**) was hydrolyzed with aqueous HCl (6 N, 300 μL) at 110 °C and stirred for 16 h using a heating block. The resulting hydrolysate was dried under a stream of air, treated with a solution of l-FDAA (1-fluoro-2–4-dinitrophenyl-5-l-alanine amide) in acetone (1.1 mL, 1 mg/mL), followed by the addition of aqueous NaHCO_3_ (1 M, 300 μL). The mixture was stirred and heated to 40 °C for 1 h using a heating block. The reaction was quenched with aqueous HCl (1 M, 300 μL), dried under nitrogen and resuspended in 100 μL 50% CH_3_CN/H_2_O for LR-LCMS analysis. Authentic amino acid-FDAA derivative standards were prepared using a similar method to the hydrolysate product. Derivatized standards and hydrolysate were analyzed by reversed-phase LCMS equipped with a Synergi Hydro-RP column (4 μm, 250 × 4.6 mm), DAD, and MS (LTQ) detectors under the following conditions: 0.3 mL/min, hold 20% CH_3_CN + 0.1% FA/80% H_2_O + 0.1% FA for 5 min then gradient to 42.3% CH_3_CN + 0.1% FA/57.7% H_2_O + 0.1% FA over 130 min. Retention times of the derivatized standards were: l-Ala (63.5 min), d-Ala (77.3 min), l-Val (95 min) d-Val (116.3 min), l-*allo*-Thr (44.5 min), d-*allo*-Thr (49.6 min), l-Thr (43.9 min), d-Thr (58.2 min), l-Phe (120.5 min), d-Phe (136.3), l-Leu (119.6 min), d-Leu (140 min). The derivatized hydrolysate product of **1** had peaks matching l-Ala, l-Val, d-*allo*-Thr, both l-Phe, d-Phe, and d-Leu. The extracted ion chromatograms (EIC) were generated using smoothing with Gaussian 11 and plotted with GraphPad Prism (v10.4.1).

### Mass Spectrometry Metabolomics of Biofilm Inhibition Fractions

The six biofilm inhibiting fractions produced by Puerto Rico Strains were resuspended in 50% CH_3_OH/H_2_O at a concentration of 0.5 mg/mL and analyzed by LR-LCMS for the presence of **1**–**8**. The LC was equipped with a Phenomenex Kinetex 5 μm C8 (100 × 3.0 mm) column and run under the following method: flow rate of 0.3 mL/min, hold 10% CH_3_CN + 0.1% FA/H_2_O + 0.1% FA for 3 min then gradient to 100% CH_3_CN + 0.1% FA over 14 min. The compounds of interest eluted between 12 to 15 min and were present in crude extracts—EM729, EM740, EM737, EM762, and EM739.

### Mass Spectrometry Metabolomics on the Fraction Library

Each fraction was resuspended in 50% CH_3_CN/H_2_O at a concentration of 10 mg/mL and analyzed on a Thermo Q Exactive Plus mass spectrometer equipped with an electrospray ionization (ESI) source coupled with a Waters Acquity ultraperformance liquid chromatography (UPLC). An aliquot (5 μL) of each fraction was processed using a Waters BEH C18, 1.7 μm (2.1 mm × 50 mm) under the following conditions: 0.3 mL/min, hold 10% CH_3_CN + 0.1% FA/90% H_2_O + 0.1% FA for 1.5 min then gradient to 100% CH_3_CN + 0.1% FA over 9.5 min. Analysis was conducted in full scan acquisition, collecting profile data in positive polarity. MS1 scan range was from 250 to 1200 *m*/*z* with a scan time of 100 ms. MS2 data were collected from 200 to 2000 *m*/*z*, with an intensity threshold of 1.6 × 10^5^ and a minimum AGC target of 8.00 × 10^3^. Dynamic exclusion was enabled where the same parent ion was chosen twice prior to being omitted for 120 s. The resolution for the MS1 scans was set to 70,000, while the MS2 scans were acquired at a resolution of 17,500. The analysis was conducted in data-dependent acquisition (DDA) mode with eight MS2 scans per MS1 scan.

### Global Natural Product Social (GNPS) Molecular Networking on Fraction Library

The raw MS files were converted into mzML by MSConvert^41^ and analyzed on the GNPS platform. A molecular network was created using the online workflow (https://ccms-ucsd.github.io/GNPSDocumentation/) on the GNPS Web site (http://gnps.ucsd.edu).^22^ The data was filtered by removing all MS/MS fragment ions within ±17 Da of the precursor *m*/*z*. MS/MS spectra were window-filtered by choosing only the top 6 fragment ions in the ±50 Da window throughout the spectrum. The precursor ion mass tolerance was set to 1.0 Da, and an MS/MS fragment ion tolerance of 0.5 Da. A network was then created where edges were filtered to have a cosine score above 0.7 and more than 6 matched peaks. Further, edges between two nodes were kept in the network if and only if each of the nodes appeared in each other’s respective top 10 most similar nodes. Finally, the maximum size of a molecular family was set to 100, and the lowest-scoring edges were removed from molecular families until the molecular family size was below this threshold. The spectra in the network were then searched against GNPS’ spectral libraries. The library spectra were filtered in the same manner as the input data. All matches kept between network spectra and library spectra were required to have a score above 0.7 and at least 6 matched peaks. The resulting molecular network was imported to Cytoscape^42^ for visualization.

## Supplementary Material



## Data Availability

GNPS molecular network created can be found at https://gnps.ucsd.edu/ProteoSAFe/status.jsp?task=faba760faf3944ef828a93b74b30d6c0. All HR-MSMS data used for molecular networking is available within the public MassIVE data set MSV000097361.

## Notes

NMR spectra, MS/MS fragmentation, Marfey’s analysis, additional biological assay data, media recipes and details on bacterial fraction library (PDF)
