# Supplementary material for: The Nobilamides: Potent Biofilm Inhibitors Produced by the Microbiota of Moon Snail Egg Masses
Source: ACS Omega. 2025 Jun 20;10(25):26791–8. doi: 10.1021/acsomega.5c01534 (PMC12223873; doi:10.1021/acsomega.5c01534)
Supplement: Supplementary file 1 [file ao5c01534_si_001.pdf]

**Supporting Information**

# The Nobilamides: Potent Biofilm Inhibitors Produced by the Microbiota of Moon Snail Egg Masses

*Lois Kyei,*[†] *Rose Campbell,*[†] *Carla Menegatti,*[†] *Emily Mevers*[†,*]

[†]Department of Chemistry, Virginia Tech, 1040 Drillfield Dr. MC0212, Blacksburg, Virginia, USA

*email: emevers@vt.edu; phone: 540-231-6570

TABLE OF CONTENTS

**Supplementary Figures**

**Figure S1:** Biofilm formation inhibition properties of chemical fractions…………………… 3
**Figure S2:** GNPS Network output……………………………………………………... 4
**Figure S3:** $^{1}$H NMR Spectra (600 MHz, $CD_3OD$) of A-3302-B (**1**)..........................................5
**Figure S4:** gHSQC Spectra ($^{1}$H 600 MHz, $CD_3OD$) of A-3302-B (**1**).......................................5
**Figure S5:** HMBC Spectra ($^{1}$H 600 MHz, $CD_3OD$) of A-3302-B (**1**).........................................6
**Figure S6:** ESI-MS/MS spectrum of A-3302-B (**1**)....................................................................7
**Figure S7:** ESI-MS/MS spectrum of A-3302-A (**2**)....................................................................7
**Figure S8:** ESI-MS/MS spectrum of nobilamide A (**3**)............................................................... 8
**Figure S9:** ESI-MS/MS spectrum of nobilamide B (**4**)...............................................................8
**Figure S10:** Marfey's analysis of A-3302-B (**1**)……………......................................................9
**Figure S11:** Marfey's analysis of A-3302-B (**1**)……………......................................................9
**Figure S12:** Marfey's analysis of A-3302-B (**1**)……………......................................................9
**Figure S13:** Marfey's analysis of A-3302-B (**1**)……………....................................................10
**Figure S14:** Marfey's analysis of A-3302-B (**1**)……………....................................................10
**Figure S15:** ESI-MS/MS spectrum of nobilamide J (**5**).............................................................11
**Figure S16:** ESI-MS/MS spectrum of nobilamide K(**6**).............................................................11
**Figure S17:** ESI-MS/MS spectrum of nobilamide S (**7**)............................................................12
**Figure S18:** ESI-MS/MS spectrum of nobilamide X (**8**)..........................................................12
**Figure S19:** Biofilm inhibition activity of A-3302-B with a positive control…..……….......13
**Figure S20:** Dose-dependent biofilm inhibition assay with alizarin…………………….....13
**Figure S21:** Biofilm inhibition activity of nobilamide A (**3**)....................................................14
**Figure S22:** Biofilm inhibition activity of nobilamide B (**4**).................................................... 14
**Figure S23:** Biofilm disruption activity of A-3302-B (**1**)........................................….15
**Figure S24:** Biofilm disruption activity of A-3302-A (**2**)........................................................15

**Supplementary Tables**

**Table S1:** Bacterial strains isolated from Western Puerto Rico………………………………16
**Table S2:** Media recipes…………………………………………………………………22
**Table S3.** ΔΔ of $^{13}$C NMR Data for A-3302-B (**1**)……………………………………………23

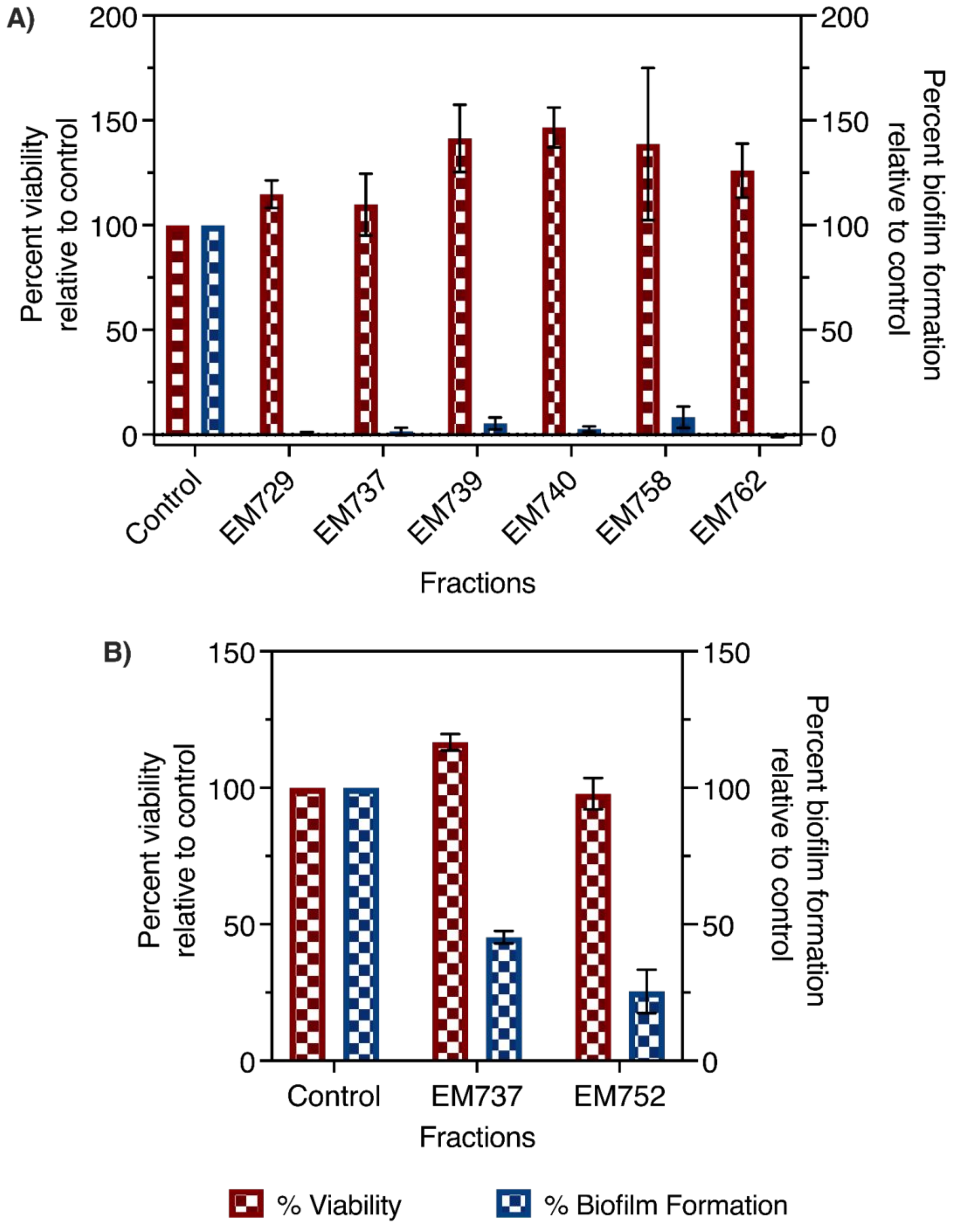


**Figure S1:** Biofilm formation inhibition properties of chemical fractions from bacteria associated with moon snail egg masses collected in Combate Beach, Puerto Rico. Activity against **(A)** *S. aureus* and **(B)** *P. aeruginosa*

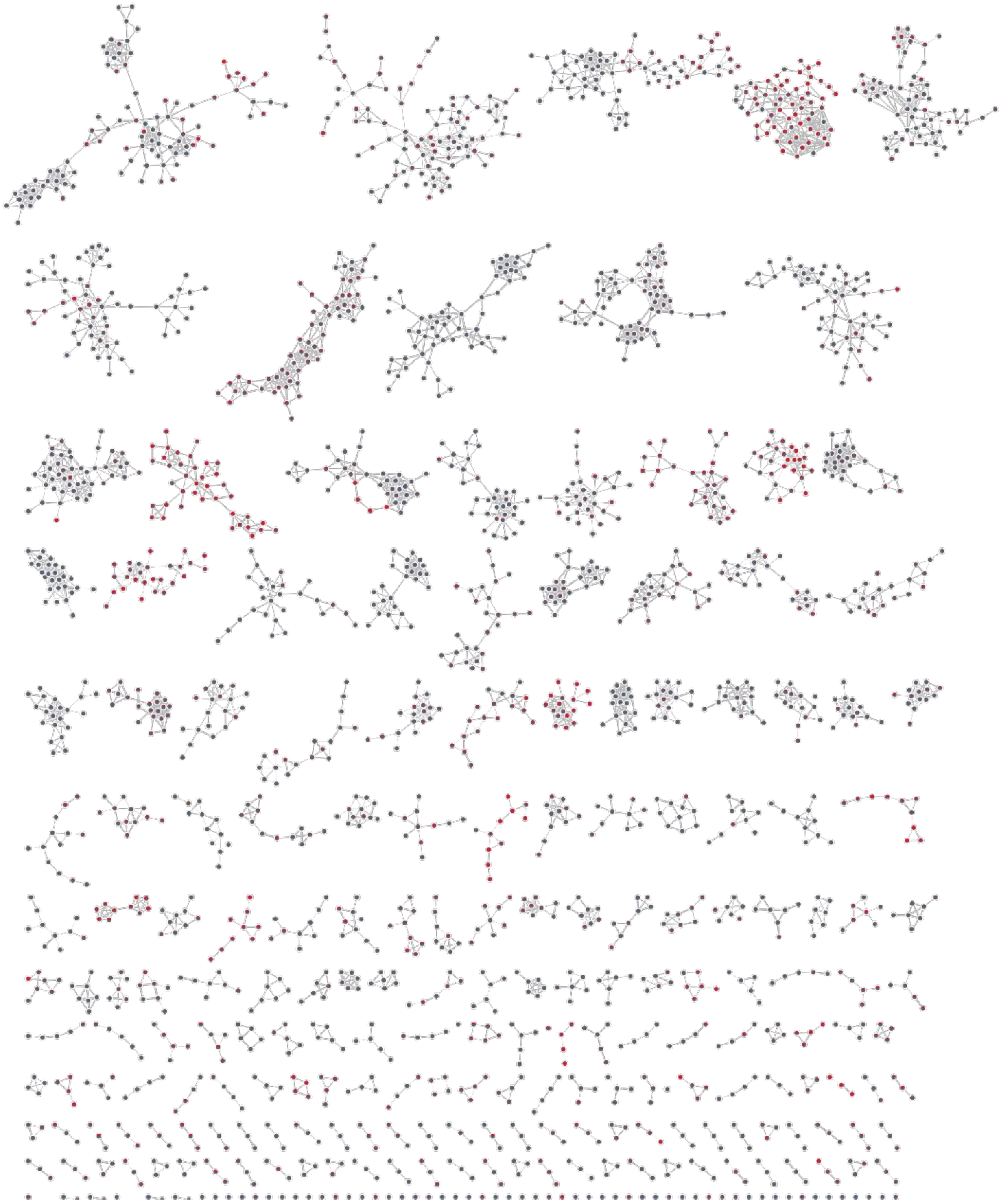

**Figure S2:** GNPS Network output excluding single nodes

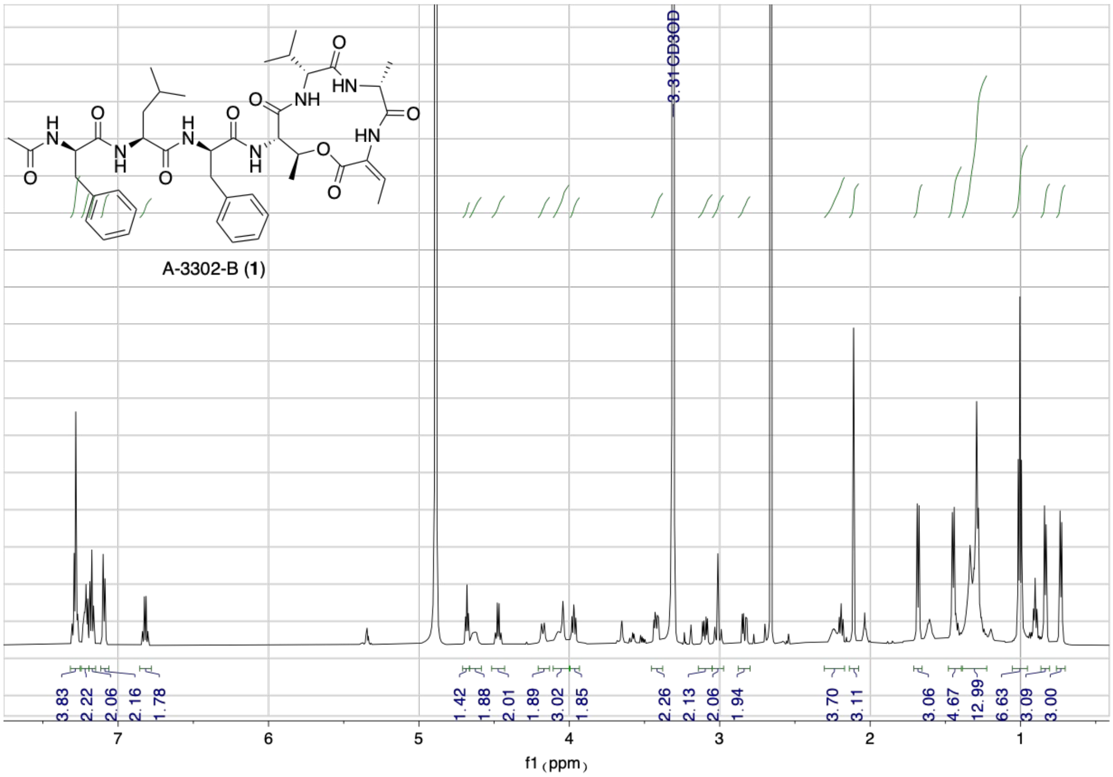

**Figure S3:** $^{1}$H NMR Spectra (600 MHz, $CD_3OD$) of A-3302-B (**1**). 256 scans.

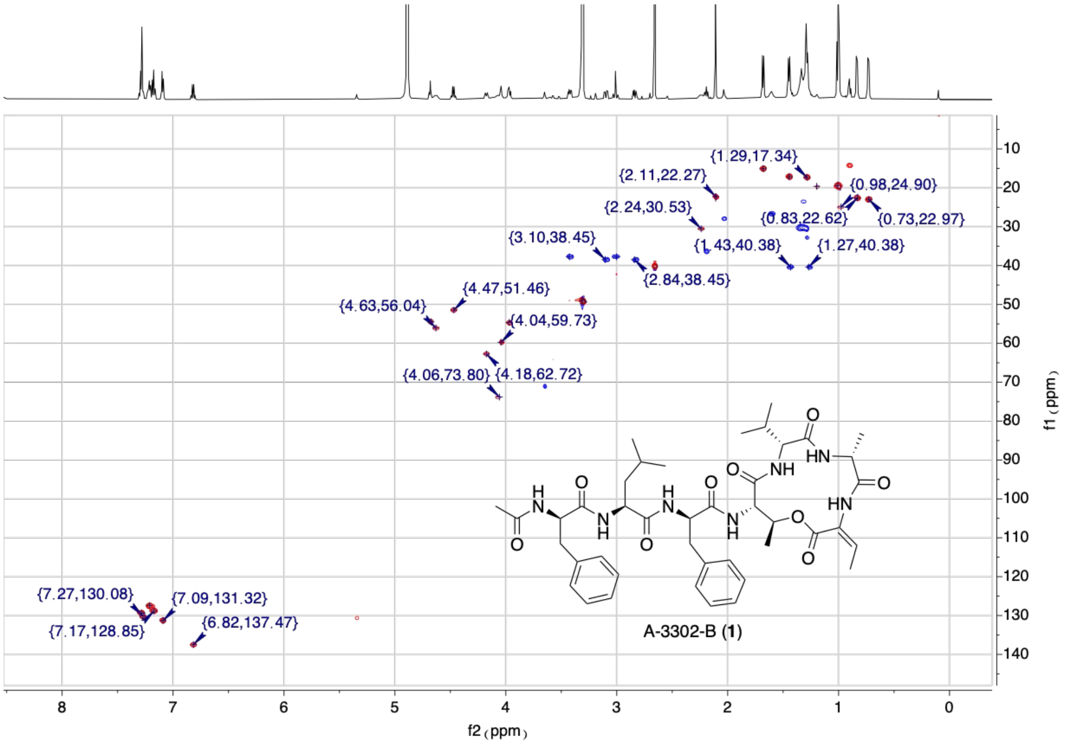

**Figure S4:** gHSQC Spectra ($^{1}$H 600 MHz, $CD_3OD$) of A-3302-B (**1**). NUS25, 256 scans.

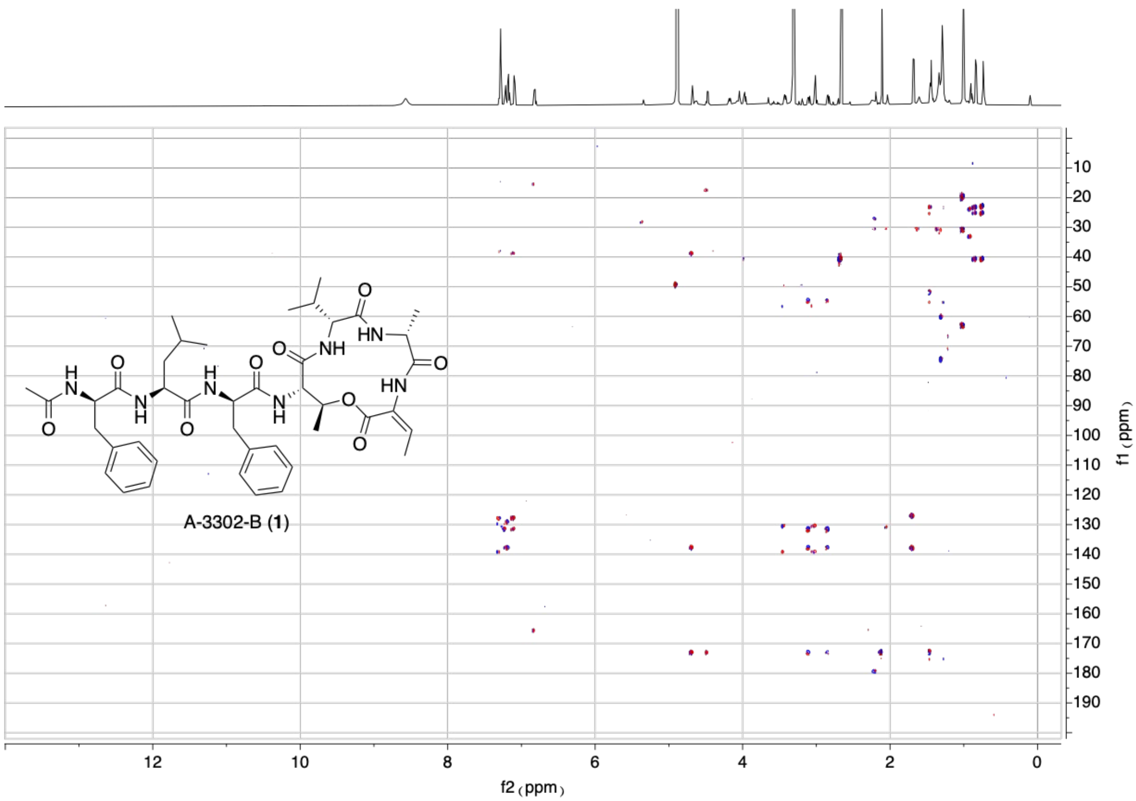


**Figure S5:** HMBC Spectra ($^{1}H$ 600 MHz, $CD_3OD$) of A-3302-B (**1**). NUS50, 256 scans

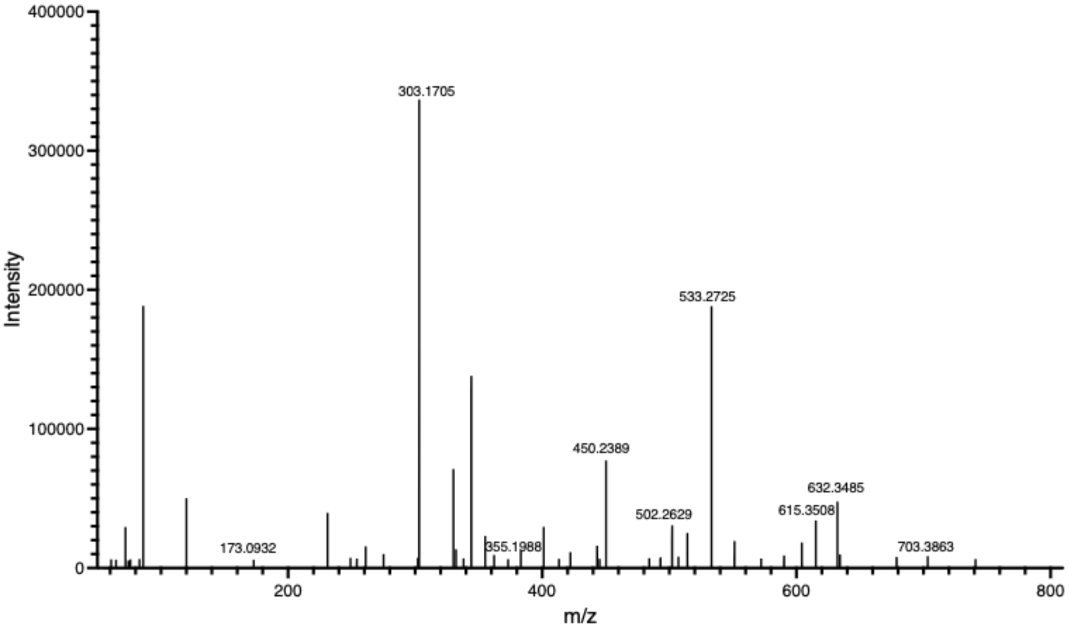


**Figure S6.** ESI-MS/MS spectrum of A-3302-B (**1**) showing y- and b-ion fragments.

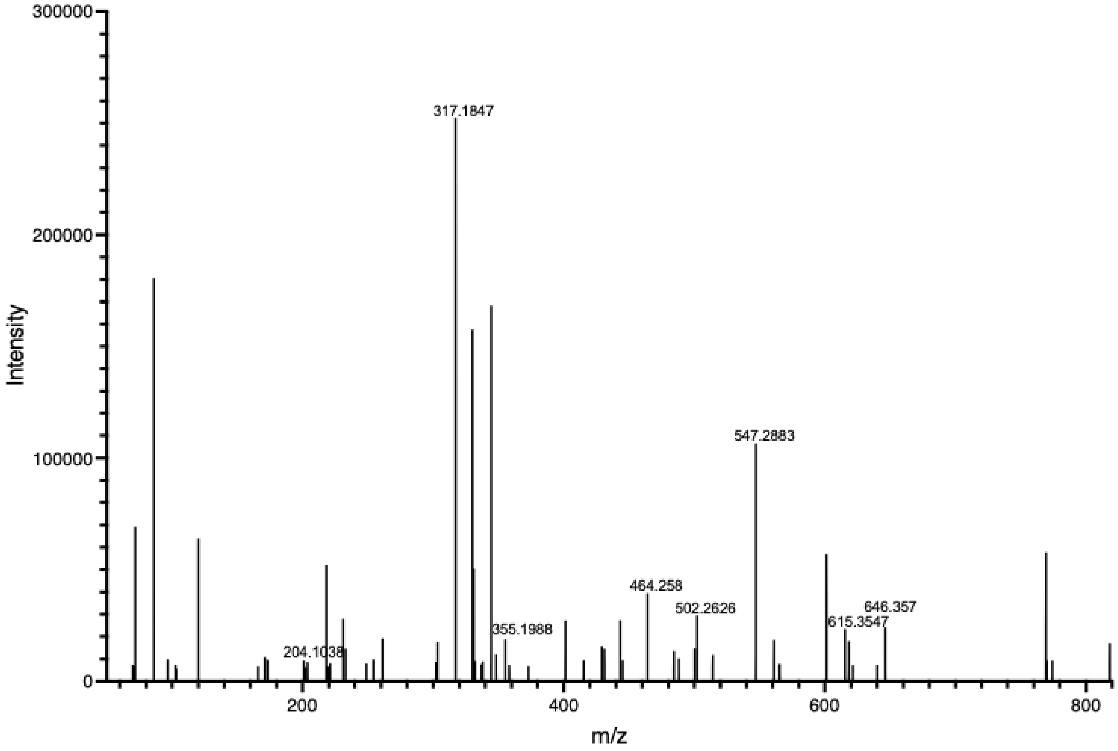


**Figure S7.** ESI-MS/MS spectrum of A-3302-A (**2**) showing y- and b-ion fragments.

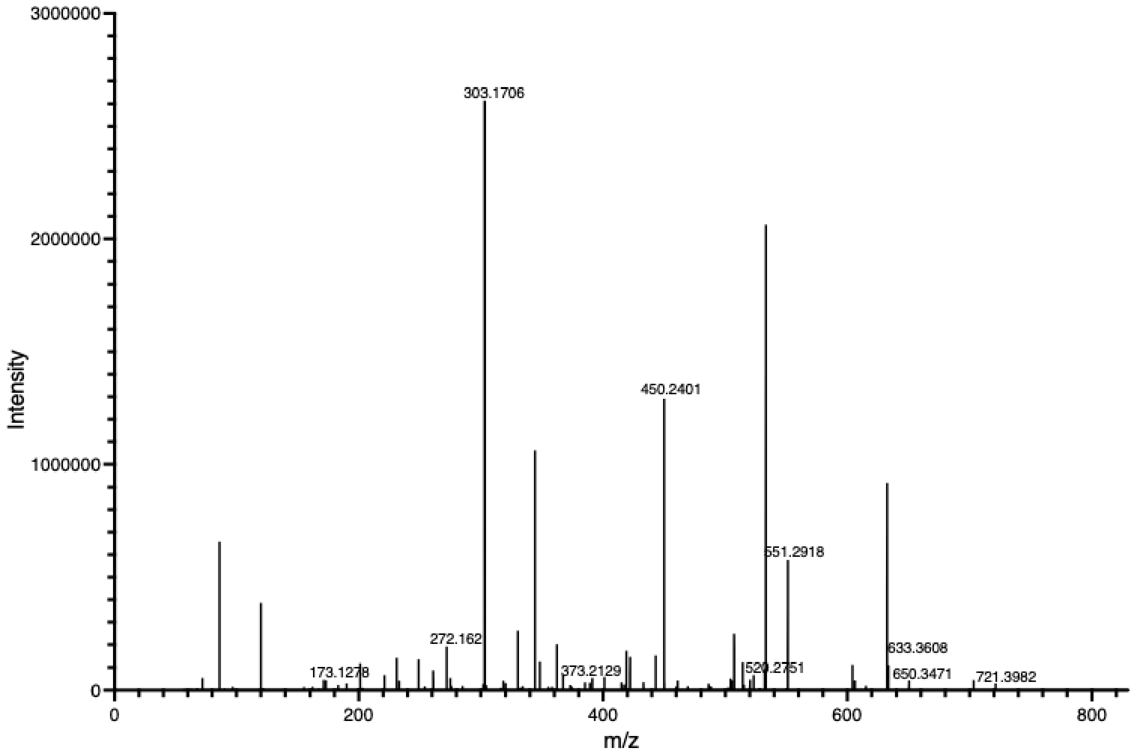


**Figure S8.** ESI-MS/MS spectrum of nobilamide A (**3**) showing y- and b-ion fragments.

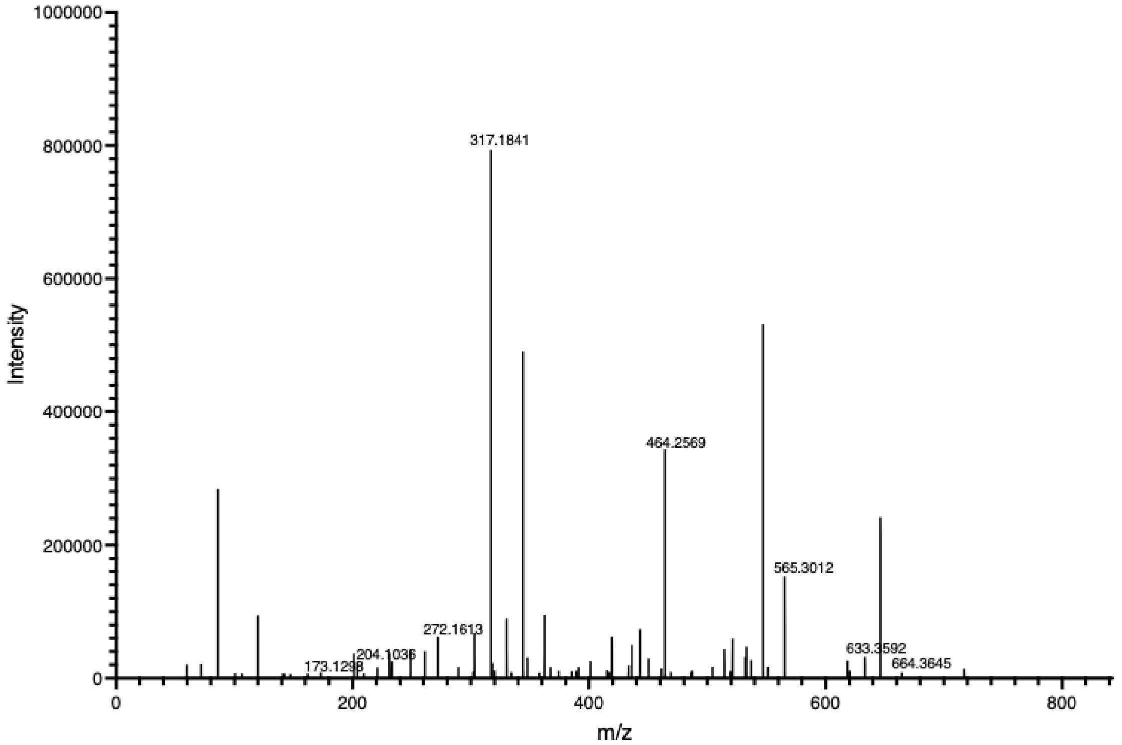


**Figure S9.** ESI-MS/MS spectrum of nobilamide B (**4**) showing y- and b-ion fragments.

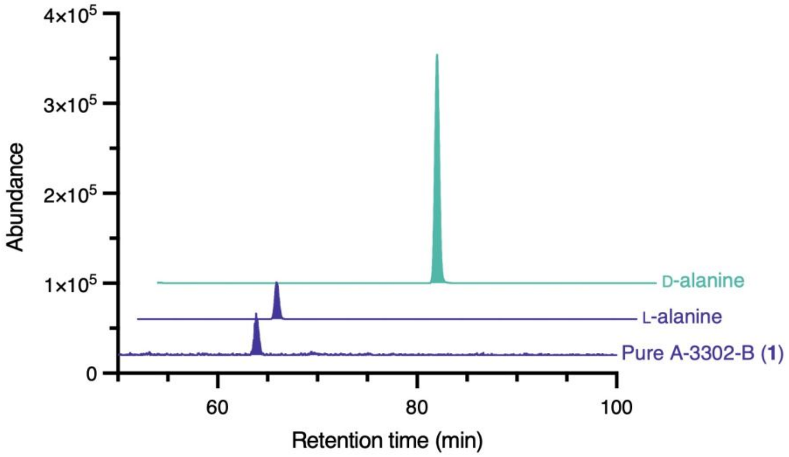


**Figure S10.** Marfey's analysis of pure A-3302-B (**1**) indicating the presence of L-alanine.

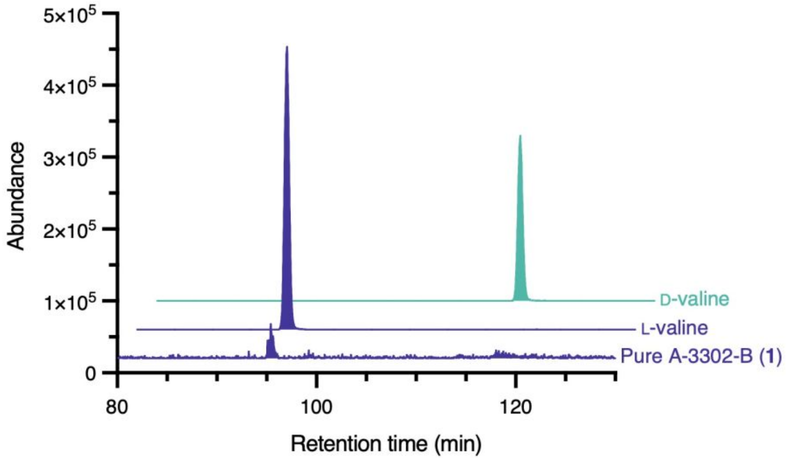


**Figure S11.** Marfey's analysis of pure A-3302-B (**1**) indicating the presence of L-valine.

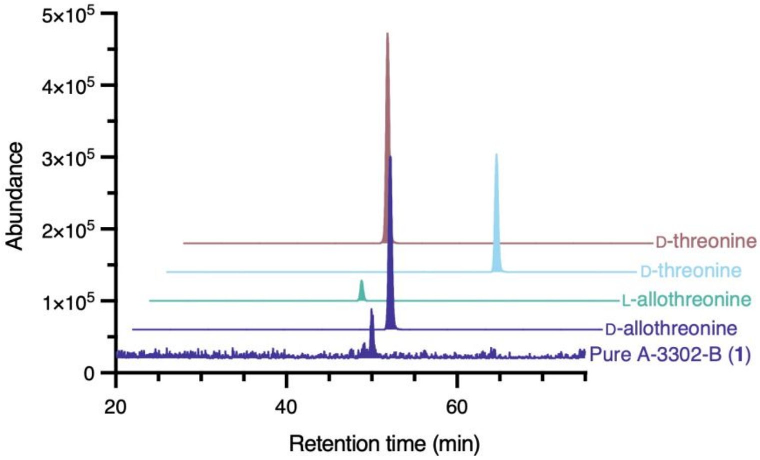


**Figure S12.** Marfey's analysis of pure A-3302-B (**1**) indicating the presence of D-*allo*-threonine.

D) 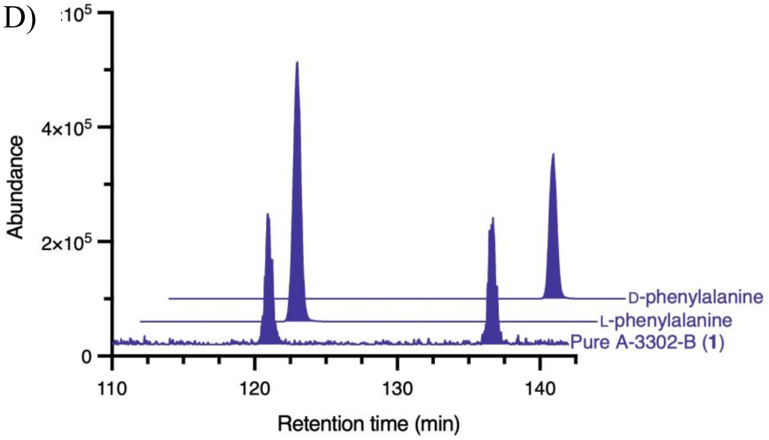


**Figure S13.** Marfey's analysis of pure A-3302-B (**1**) indicating the presence of both L- and D-phenylalanine.

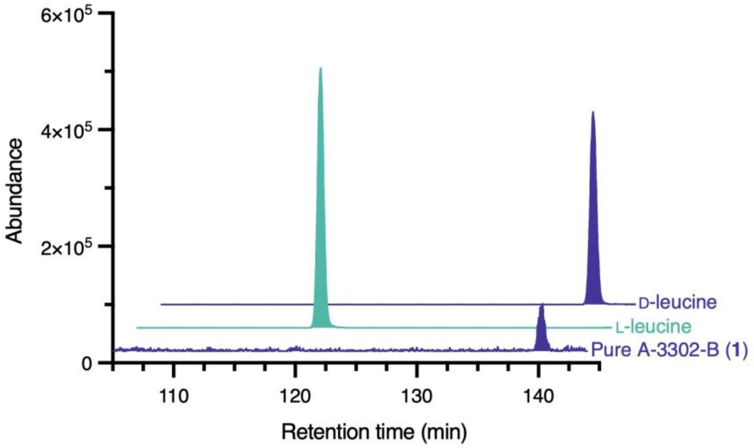


**Figure S14.** Marfey's analysis of pure A-3302-B (**1**) indicating the presence of D-leucine.

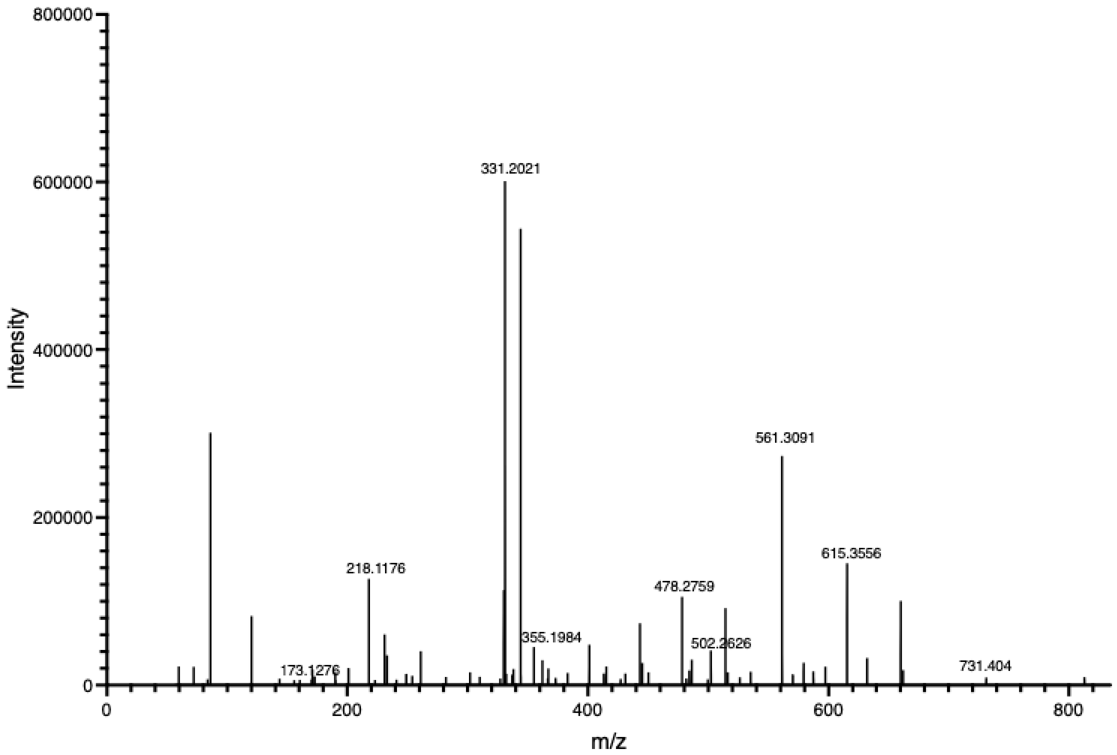


**Figure S15.** ESI-MS/MS spectrum of nobilamide J (**5**) showing y- and b-ion fragments.

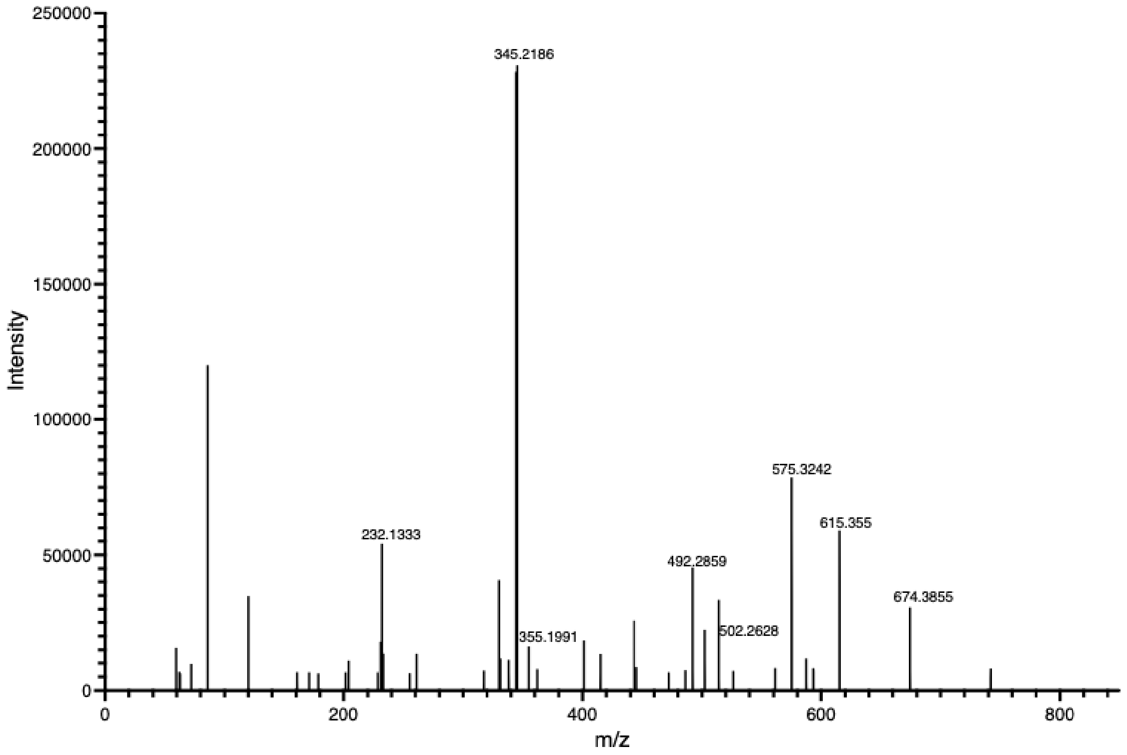


**Figure S16.** ESI-MS/MS spectrum of nobilamide K (**6**) showing y- and b-ion fragments.

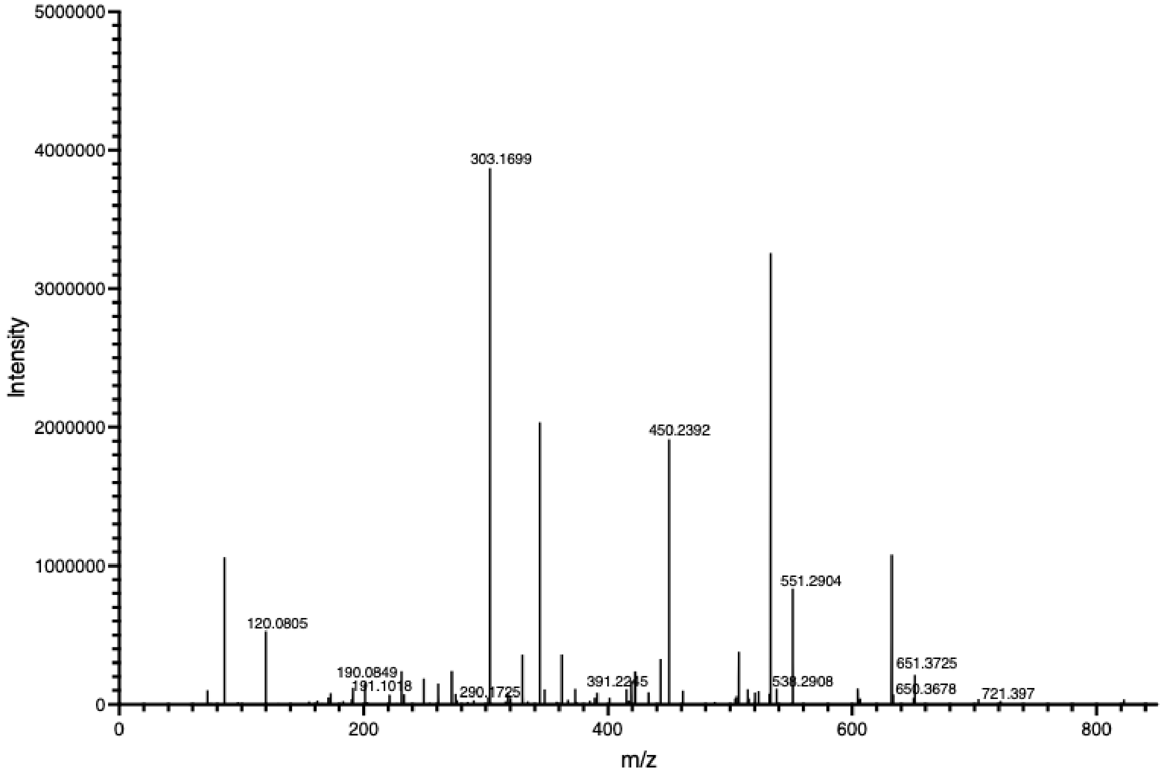


**Figure S17.** ESI-MS/MS spectrum of nobilamide S (**7**) showing y- and b-ion fragments.

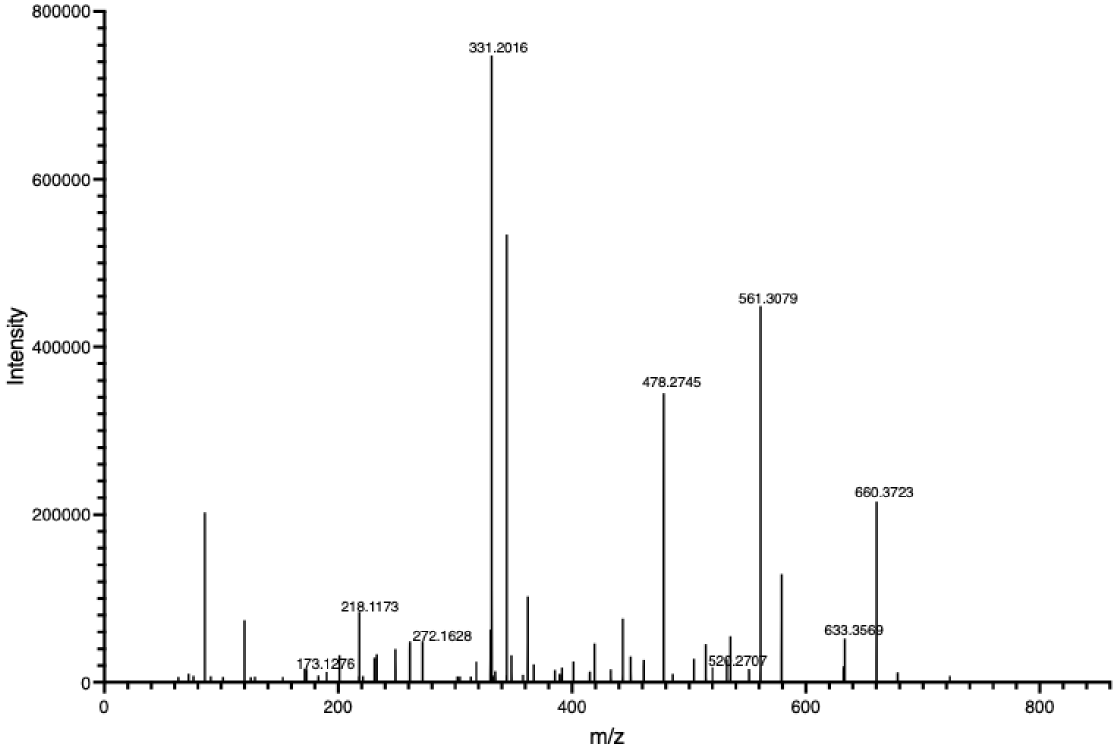


**Figure S18.** ESI-MS/MS spectrum of nobilamide X (**8**) showing y- and b-ion fragments.

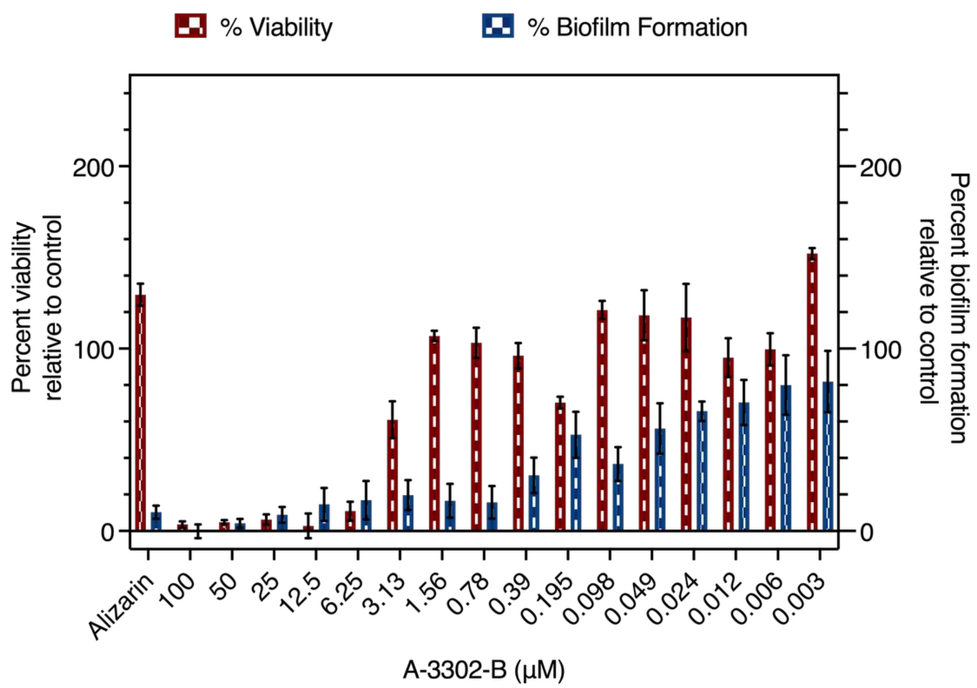


**Figure S19.** Biofilm inhibition activity of A-3302-B (**1**) in comparison to alizarin (positive control).

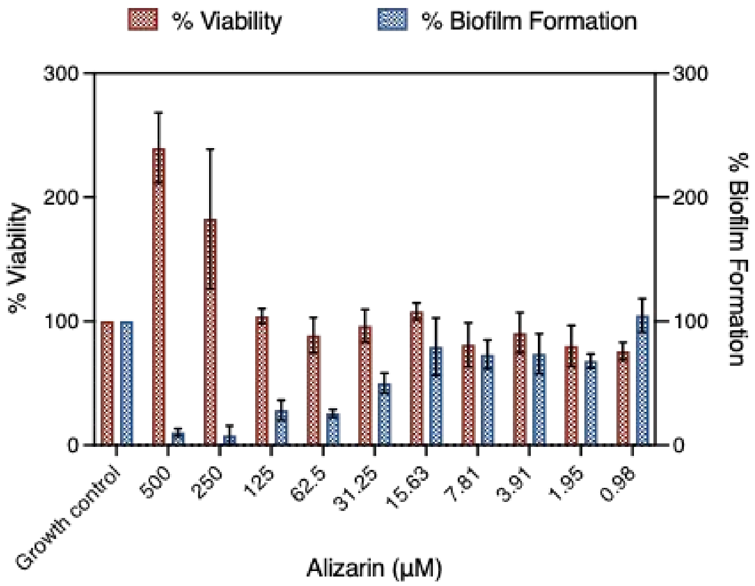


**Figure S20:** Dose-dependent biofilm inhibition assay with alizarin

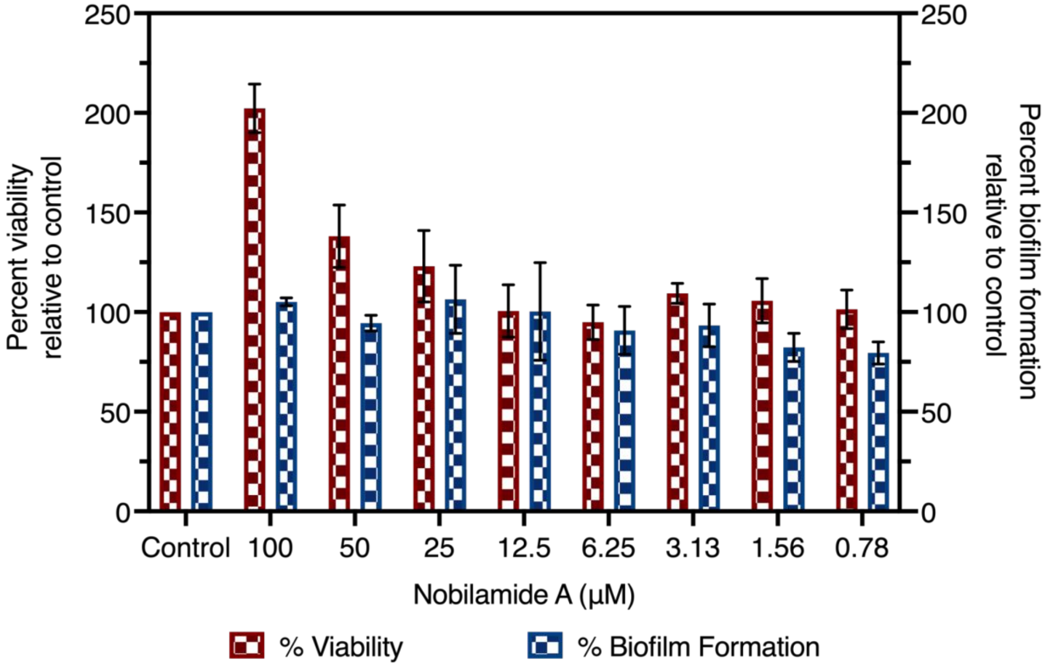


**Figure S21.** Biofilm inhibition activity of nobilamide A (**3**) against *S. aureus*.

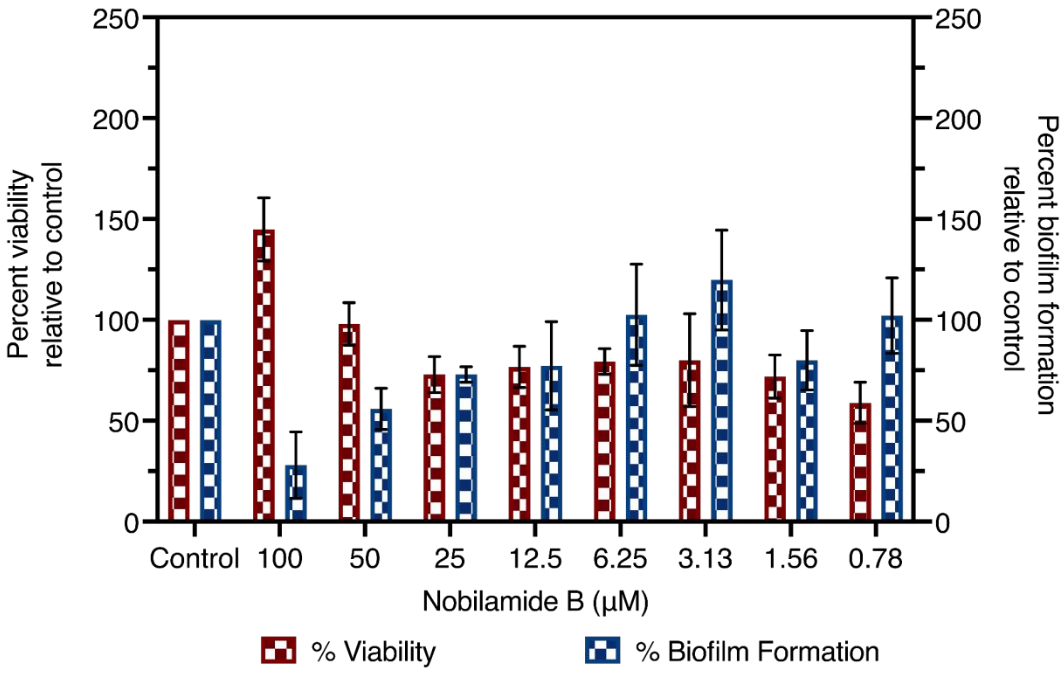


**Figure S22.** Biofilm inhibition activity of nobilamide B (**4**) against *S. aureus*.

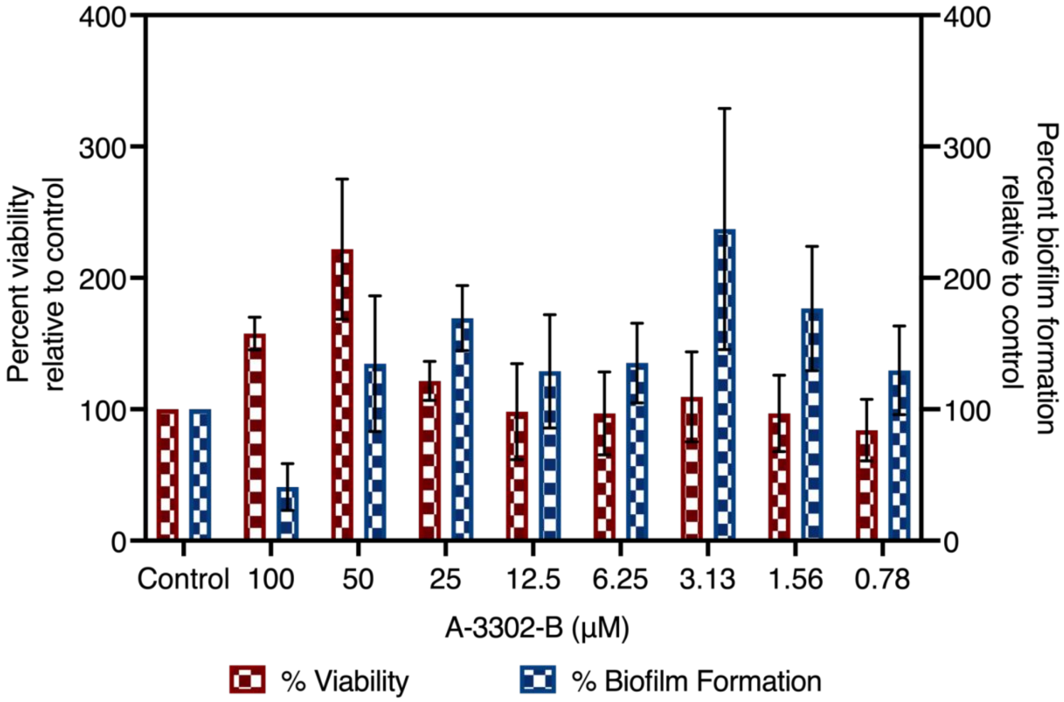


**Figure S23.** Biofilm disruption activity of A-3302-B (**1**) against *S. aureus*.

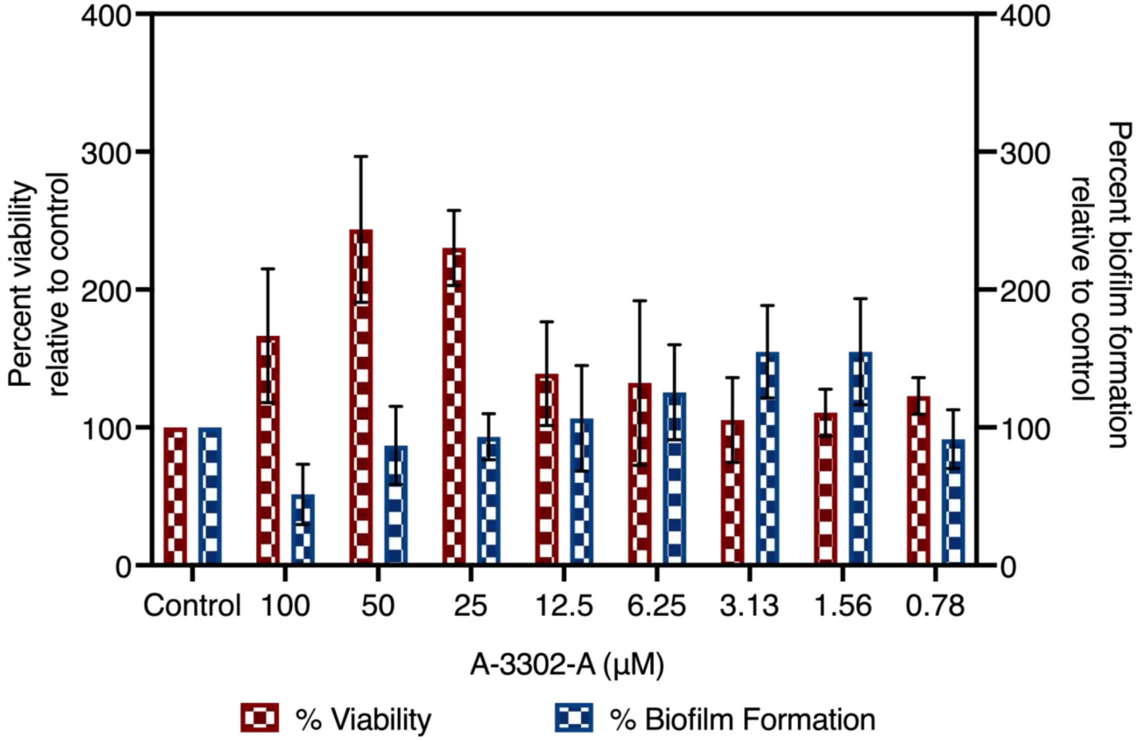


**Figure S24.** Biofilm disruption activity of A-3302-A (**2**) against *S. aureus*.

| **Table S1:** Bacterial strains isolated from Western Puerto Rico | | | |
|---|---|---|---|
| **Strain** | **Fraction code** | **Biofilm Inhibition** | |
| | | ***S. aureus*** | ***P. aeruginosa*** |
| *Paenibacillus* sp. (EM725) | EM725-A | No | No |
| | EM725-B | | |
| *Lelliottia* sp. (EM726) | EM726-A | No | No |
| | EM726-B | | |
| *Bacillus* sp. (EM727) | EM727-A | No | No |
| | EM727-B | | |
| *Bacillus* sp. (EM728) | EM728-A | No | No |
| | EM728-B | | |
| *Exiguobacterium* sp. (EM729) | EM729-A | Yes | No |
| | EM729-B | | |
| *Exiguobacterium* sp. (EM730) | EM730-A | No | No |
| | EM730-B | | |
| *Kosakonia* sp. (EM731) | EM731-A | No | No |
| | EM731-B | | |
| *Microbacterium* sp. (EM732) | EM732-A | No | No |
| | EM732-B | | |
| *Bacillus* sp. | EM733-A | | |

| | | | |
|---|---|---|---|
| (EM733) | EM733-B | No | No |
| *Aurantiacibacter* sp. (EM734) | EM734-A<br>EM734-B | No | No |
| *Alkalihalobacillus* sp. (EM735) | EM735-A<br>EM735-B | No | No |
| *Aureimonas* sp. (EM736) | EM736-A<br>EM736-B | No | No |
| *Staphylococcus* sp. (EM737) | EM737-A<br>EM737-B | Yes | Yes |
| *Microbacterium* sp. (EM738) | EM738-A<br>EM738-B | No | No |
| Unknown (EM739) | EM739-A<br>EM739-B | Yes | No |
| *Bacillus* sp. (EM740) | EM740-A<br>EM740-B | Yes | No |
| Unknown (EM741) | EM741-A<br>EM741-B | No | No |
| Unknown (EM742) | EM742-A<br>EM742-B | No | No |
| *Rhodobacter* sp. | EM743-A | | |

| | | | |
|---|---|---|---|
| (EM743) | EM743-B | No | No |
| *Curtobacterium* sp.(EM744) | EM744-A | No | No |
| | EM744-B | | |
| *Microbacterium* sp. (EM745) | EM745-A | No | No |
| | EM745-B | | |
| *Curtobacterium* sp. (EM746) | EM746-A | No | No |
| | EM746-B | | |
| *Cytobacillus* sp. (EM747) | EM747-A | No | No |
| | EM747-B | | |
| *Pseudomonas* sp. (EM748) | EM748-A | No | No |
| | EM748-B | | |
| *Bacillus* sp. (EM749) | EM749-A | No | No |
| | EM749-B | | |
| Unknown (EM750) | EM750-A | No | No |
| | EM750-B | | |
| *Paenibacillus* sp. (EM751) | EM751-A | No | No |
| | EM751-B | | |
| *Bacillus* sp. (EM752) | EM752-A | No | Yes |
| | EM752-B | | |
| *Acinetobacter* sp. (EM753) | EM753-A | No | No |
| | EM753-B | | |

| | | | |
|---|---|---|---|
| Unknown (EM754) | EM754-A<br>EM754-B | No | No |
| *Exiguobacterium* sp. (EM755) | EM755-A<br>EM755-B | No | No |
| *Curtobacterium* sp. (EM756) | EM756-A<br>EM756-B | No | No |
| *Bacillus* sp. (EM757) | EM757-A<br>EM758-B | No | No |
| Unknown (EM758) | EM758-A<br>EM758-B | Yes | No |
| *Bacillus* sp. (EM759) | EM759-A<br>EM759-B | No | No |
| *Curtobacterium* sp. (EM760) | EM760-A<br>EM760-B | No | No |
| *Staphylococcus* sp. (EM761) | EM761-A<br>EM761-B | No | No |
| *Staphylococcus* sp. (EM762) | EM762-A<br>EM762-B | Yes | No |
| Unknown (EM763) | EM763-A<br>EM763-B | No | No |

| | | | |
|---|---|---|---|
| *Streptomyces* sp. (EM764) | EM764-A | No | No |
| | EM764-B | | |
| Unknown (EM765) | EM765-A | No | No |
| | EM765-B | | |
| Unknown (EM766) | EM766-A | No | No |
| | EM766-B | | |
| Unknown (EM767) | EM767-A | No | No |
| | EM767-B | | |
| *Rossellomorea* sp. (EM768) | EM768-A | No | No |
| | EM768-B | | |
| *Fictibacillus* sp. (EM769) | EM769-A | No | No |
| | EM769-B | | |
| *Bacillus* sp. (EM770) | EM770-A | No | No |
| | EM770-B | | |
| *Ruegeria* sp. (EM771) | EM771-A | No | No |
| | EM771-B | | |
| *Ruegeria* sp. (EM772) | EM772-A | No | No |
| | EM772-B | | |
| *Ruegeria* sp. (EM773) | EM773-A | No | No |
| | EM773-B | | |

| | | | |
|---|---|---|---|
| *Ruegeria* sp. (EM774) | EM774-A | No | No |
| | EM774-B | | |
| *Micromonospora* sp. (EM775) | EM775-A | No | No |
| | EM775-B | | |
| *Oceanobacillus* sp. (EM776) | EM776-A | No | No |
| | EM776-B | | |
| *Ruegeria* sp. (EM777) | EM777-A | No | No |
| | EM777-B | | |

| **Table S2:** Media Recipes | | | | |
|---|---|---|---|---|
| YEME | R2A | A-media | M63 | Artificial Seawater |
| 4 g yeast extract | 0.5 g acicase | 20 g starch | 2 g $(NH_4)SO_4$ | 1:1 mixture of solution A and B |
| 10 g malt extract | 0.5 g yeast extract | 5 g peptone | 13.6 g $KH_2PO_4$ | **Solution A** |
| 4 g dextrose | 0.5 g proteose peptone | 10 g dextrose | 0.5 mg $FeSO_4.7H_2O$ | 415.2 g NaCl |
| 20 g Agar | 0.5 g dextrose | 5 g yeast | 1 mL of 1M $MgSO_4.7H_2O$ | 69.5 g $Na_2SO_4$ |
| 1 L seawater | 0.5 g starch, soluble | 5 g $CaCO_3$ | 10 mL of 20% glucose (water) | 11.74 g KCl |
| | 0.3 g $K_2HPO_4$ | 1 L seawater | 0.1 mL of 0.5% thiamine | 3.4 g $NaHCO_3$ |
| | 0.024 g $MgSO_4.7H_2O$ | | 5 mL of 20% casamino acids | 1.7 g KBr |
| | 0.3 g sodium pyruvate | | 1 L MilliQ water | 0.45 g $H_3BO_3$ |
| | 1 L seawater | | | 0.054 g NaF |
| | | | | 10 L DI water |
| | | | | |
| | | | | **Solution B** |
| | | | | 187.9 g $MgCl_3.6H_2O$ |
| | | | | 22.7 g $CaCl_2.2H_2O$ |
| | | | | 0.428 g $SrCl_2.6H_2O$ |
| | | | | 10 L DI water |

| **Table S3.** ΔΔ of $^{13}$C NMR Data for A-3302-B (**1**) | | |
|---|---|---|
| Reported $^{13}$C NMR (ppm)* | $^{13}$C NMR from this study (ppm) | Δreported-this study (ppm) |
| 175.6 | 175.1 | 0.5 |
| 174.3 | 173.3 | 1 |
| 173.9 | 173.3 | 0.6 |
| 173.3 | 173.2 | -0.1 |
| 165.0 | 165.4 | -0.4 |
| 138.9 | 139.0 | -0.9 |
| 138.1 | 139.0 | -0.1 |
| 137.6 | 137.6 | 0 |
| 137.2 | 137.7 | -0.5 |
| 136.6 | 137.2 | -0.6 |
| 130.7 | 131.3 | -0.6 |
| 130.5 | 130.3 | 0.2 |
| 130.3 | 129.4 | 0.9 |
| 129.6 | 129.4 | 0.2 |
| 129.5 | 128.8 | 0.7 |
| 129.4 | 128.8 | 0.6 |
| 127.9 | 127.6 | 0.3 |
| 127.4 | 127.6 | -0.2 |
| 75.8 | 74.1 | 1.7 |
| 62.5 | 62.8 | -0.3 |
| 58.5 | 59.8 | -1.3 |
| 57.5 | 56.1 | 1.4 |
| 55.2 | 54.5 | 0.7 |
| 55.2 | 54.5 | 0.7 |
| 50.6 | 51.5 | -0.9 |
| 40.9 | 40.5 | 0.4 |
| 38.1 | 38.7 | -0.6 |
| 36.9 | 37.9 | -1 |
| 30.6 | 30.5 | 0.1 |
| 25.5 | 24.9 | 0.6 |
| 23.0 | 23.0 | 0 |
| 22.6 | 22.7 | -0.1 |
| 22.3 | 22.4 | -0.1 |
| 21.0 | ND | |
| 20.1 | 19.8 | 0.3 |
| 19.5 | 19.4 | 0.1 |
| 18.5 | 17.3 | 1.2 |
| 16.8 | 17.2 | -0.4 |
| 15.3 | 15.1 | 0.2 |

*from DOI: 10.3390/biom14101244
